# Improved Antiglioblastoma Activity and BBB Permeability by Conjugation of Paclitaxel to a Cell‐Penetrative MMP‐2‐Cleavable Peptide

**DOI:** 10.1002/advs.202001960

**Published:** 2020-12-21

**Authors:** Dan Hua, Lida Tang, Weiting Wang, Shengan Tang, Lin Yu, Xuexia Zhou, Qian Wang, Cuiyun Sun, Cuijuan Shi, Wenjun Luo, Zhendong Jiang, Huining Li, Shizhu Yu

**Affiliations:** ^1^ Department of Neuropathology Tianjin Neurological Institute Tianjin Medical University General Hospital Tianjin 300052 China; ^2^ Tianjin Key Laboratory of Injuries Variations and Regeneration of the Nervous System Tianjin 300052 China; ^3^ Key Laboratory of Post‐trauma Neuro‐repair and Regeneration in Central Nervous System Ministry of Education Tianjin 300052 China; ^4^ Tianjin Institute of Pharmaceutical Research Tianjin 300301 China; ^5^ Tianjin Key Laboratory on Technologies Enabling Development of Clinical Therapeutics and Diagnostics (Theranostics) School of Pharmacy Tianjin Medical University Tianjin 300070 China; ^6^ Department of Biochemistry and Molecular Biology School of Basic Medical Sciences of Tianjin Medical University Tianjin 300070 China

**Keywords:** antiglioblastomas, BBB permeability, MMP‐2, paclitaxel, peptide‐drug conjugations

## Abstract

In order to solve the problems of receptor promiscuity and poor blood‐brain barrier (BBB) penetration in the treatment of glioblastomas (GBM), a novel dual‐functional nanocomplex drug delivery system is developed based on the strategy of peptide‐drug conjugates. In this study, SynB3‐PVGLIG‐PTX is designed and screened out by matrix metalloproteinase‐2 (MMP‐2), to which it exhibits the best affinity. The MMP‐2‐sensitive peptide (PVGLIG) and a cell‐penetration peptide (SynB3) are combined to form a dual‐functional peptide. Moreover, as a drug‐peptide nanocomplex, SynB3‐PVGLIG‐PTX exhibited a high potential to form an aggregation with good solubility that can release paclitaxel (PTX) through the cleavage of MMP‐2. From a functional perspective, it is found that SynB3‐PVGLIG‐PTX can specifically inhibit the proliferation, migration, and invasion of GBM cells in vitro in the presence of MMP‐2, in contrast to that observed in MMP‐2 siRNA transfected cells. Further investigation in vivo shows that SynB3‐PVGLIG‐PTX easily enters the brain of U87MG xenograft nude mice and can generate a better suppressive effect on GBM through a controlled release of PTX from SynB3‐PVGLIG‐PTX compared with PTX and temozolomide. Thus, it is proposed that SynB3‐PVGLIG‐PTX can be used as a novel drug‐loading delivery system to treat GBM due to its specificity and BBB permeability.

## Graphical abstract

SynB3‐PVGLIG‐PTX exhibits a strong affinity with MMP‐2, and it can enhance water solubility by agglomerating to form a special structure with a positive charge; a controlled release of PTX from SynB3‐PVGLIG‐PTX occurs upon cleavage of MMP‐2, implying that SynB3‐PVGLIG‐PTX has a specific cytotoxicity in GBM cells; SynB3‐PVGLIG‐PTX can effectively inhibit GBM in vitro and in vivo.

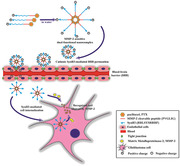

## 1 Introduction

Glioma is the most common primary intracranial tumor found in humans.^[^ ^1^, ^2^, ^3^ ^]^ Glioblastoma (GBM), a type of malignant glioma, is the most aggressive and invasive tumor in the human brain and is associated with high morbidity and mortality.^[^ ^4^, ^5^, ^6^ ^]^ Though treatment options have been significantly optimized in recent years, the conventional treatment of GBM in the clinic remains a combination of surgical resection, radiotherapy, and chemotherapy.^[^ ^2^, ^7^ ^]^ Temozolomide (TMZ) remains the most recommended chemotherapy drug for treating GBM; however, it has been unable to prolong the survival of GBM patients, who have a median survival time of only 14.6 months.^[^ ^8^, ^9^ ^]^ Meanwhile, most novel chemotherapy drugs cannot be employed to treat GBM at present, primarily because they are difficult to introduce into the central nervous system (CNS) through the circulatory system, partially due to the protection of the blood‐brain barrier (BBB).^[^ ^10^, ^11^, ^12^, ^13^, ^14^ ^]^

Peptide‐drug conjugates (PDCs), used as part of a noninvasive and safe drug delivery system, have aroused widespread attention for their advantages in the improvement of treatment efficacy, specificity, water‐solubility and biological activity, as well as for acting as vehicles for carrying almost all drugs, compared with other drug delivery approaches.^[^ ^15^, ^16^, ^17^ ^]^ To facilitate the cellular internalization and BBB penetration of chemotherapy drugs, cell‐penetrating peptides (CPPs) have been typically adopted as nanocarriers to build drug delivery systems (e.g., PDCs).^[^ ^18^, ^19^ ^]^ Furthermore, unlike other receptor transporters, CPPs penetrate the BBB through a nonsaturable influx that cannot cause a transient increase in intracerebral drug concentration.^[^ ^20^ ^]^ Thus, PDCs based on CPPs are potentially an ideal drug delivery strategy for GBM therapy. In the past two decades, hundreds of CPPs have been developed and described by scientific studies, including pVEC, TP10, TP10‐2, SynB3, and Tat 47–57. CPPs, SynB3, Tat 47–57, and pVEC cross the BBB much more easily than do TP10 and TP10‐2 on account of their different structures.^[^ ^20^, ^21^, ^22^ ^]^ Though drug delivery strategies based on CPPs can enhance BBB penetration, the application of CPPs is limited by its own nonselectivity, leading to a serious systemic toxicity in vivo.^[^ ^23^, ^24^ ^]^ To enhance the tumor‐specificity of CPPs, it is ideal to combine the tumor targeting peptides (TTPs) to form an integrated drug‐loaded nanocarrier.^[^ ^25^, ^26^, ^27^ ^]^

Matrix metalloproteinases‐2 (MMP‐2; 72kD), a member of the MMP family, plays multiple roles in the progression of tumors.^[^ ^28^, ^29^ ^]^ Compared with almost no expression in normal brain tissue, it has been suggested that MMP‐2 is highly expressed in glioma tissues.^[^ ^30^, ^31^ ^]^ Furthermore, mRNA and protein levels of MMP‐2 increase along with the stage of glioma progression, closely associated with the invasion of glioma cells.^[^ ^32^, ^33^, ^34^ ^]^ Moreover, MMP‐2 can recognize and cleave peptides with specific sequences, making it a hotspot for targeted drug release studies.^[^ ^35^ ^]^ Recent studies, including ours, have reported that the sequence of PVGLIG is a feasible matrix metalloproteinase‐sensitive linker, which has always been used as a TTP to build a targeted released drug delivery system.^[^ ^36^, ^37^ ^]^ In this study, we proposed that the MMP‐2‐sensitive linker can be combined with CPPs to form a dual‐functional peptide possessing both BBB permeability and a targeting effect and that it can also be adopted as a bridge between CPPs and drugs in the conjugate, capable of achieving targeting release of loaded‐drug.

Paclitaxel (PTX) is one of the most effective antitumoral drugs. PTX, as a cytotoxic agent, inhibits the proliferation of tumor cells through the stabilization of microtubules and blockage of the cell cycle by promoting the polymerization of tubulin proteins.^[^ ^38^ ^]^ Nevertheless, PTX is likely to cause various side effects in clinical application due to its low solubility in water and off‐target cell toxicity.^[^ ^39^, ^40^, ^41^ ^]^ To solve these issues, several delivery systems have been successfully used to load PTX, which have optimized the solubility and targeting of PTX.^[^ ^42^ ^]^ However, low BBB permeability remains a major problem for PTX in GBM treatment.

This study aimed to find a novel dual‐functional nanocomplex drug delivery system following the strategy of PDCs to treat malignant gliomas, especially GBM. Thus, a dual‐functional PDC, SynB3‐PVGLIG‐PTX, was designed, screened, and synthetized, in which PTX was combined with CPPs (e.g., SynB3) through an MMP‐2‐sensitive linker (PVGLIG). This peptide‐drug complex exhibits three advantages as follows: 1) the constructed structure of CPPs should help enhance the permeability of PTX‐containing nanocomplex across the BBB; 2) this nanocomplex covers a MMP‐2‐sensitive linker between CPPs and PTX, making it possible to release the drug at the target site with high MMP‐2 expression level; and 3) the novel dual‐functional PTX prodrug is a water‐soluble nanocomplex, which can overcome the side effect exerted by low solubility and formulating agent (Cremophore EL) of PTX.^[^ ^43^ ^]^ In conclusion, the dual‐functional drug‐loaded nanocomplex, SynB3‐PVGLIG‐PTX, is proposed here as a potentially innovative strategy for clinical treatment of GBM.

## 2 Results

### 2.1 Molecular Docking and Binding Energy Calculation

**Figure 1:**
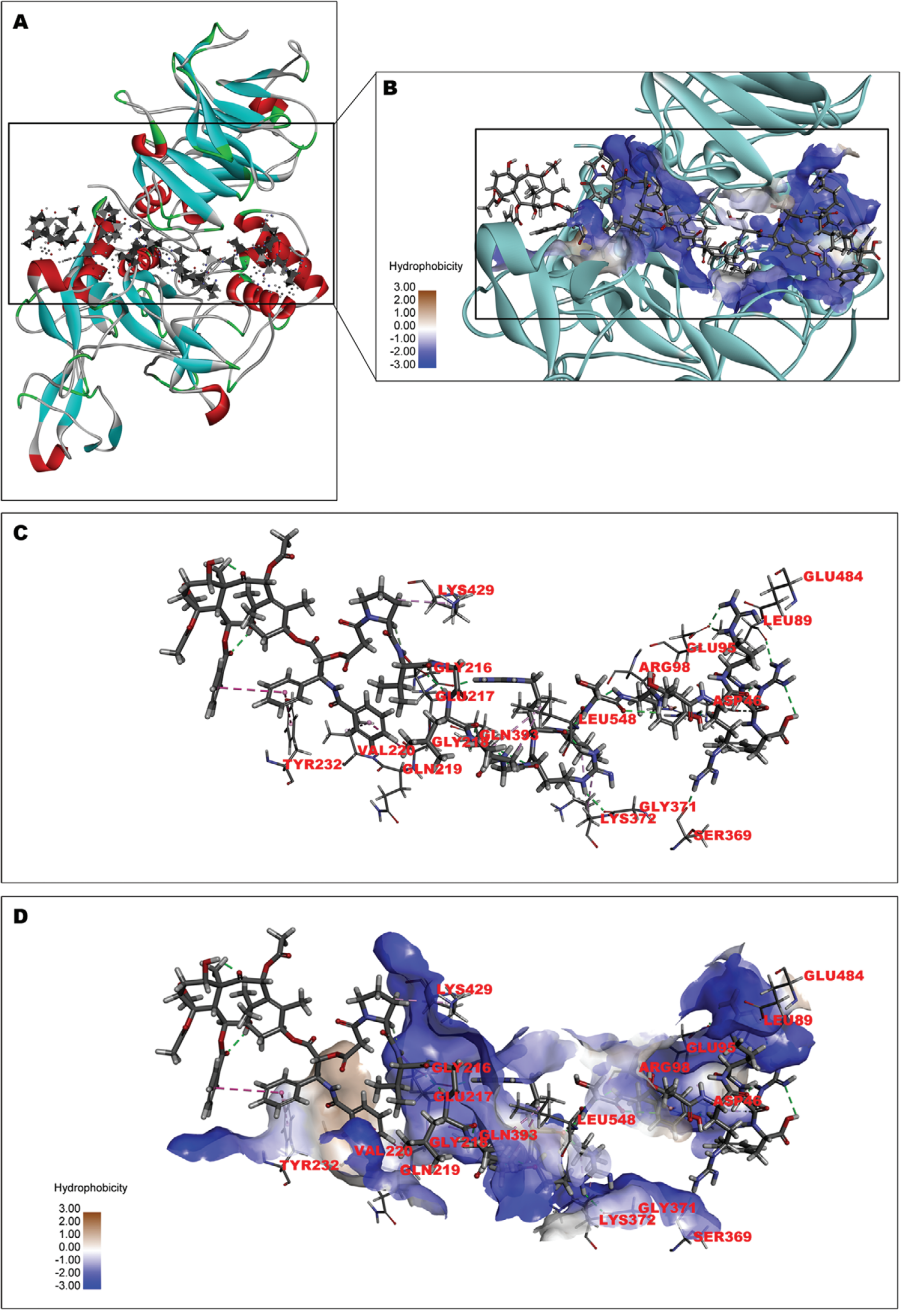
Interaction and the binding pattern of SynB3‐PVGLIG‐PTX and MMP‐2. The structure of MMP‐2 in this study was collected from the RCSB PDB (ID: 1CK7). A) The binding poses of MMP‐2 (the ribbon structure) with SynB3‐PVGLIG‐PTX (−151.29 kcal mol^−1^). In the structure of MMP‐2, *β*‐sheet (blue), loop (gray), *α*‐helix (red), and the amino acid structure connecting different segments (green) are demonstrated. The gray structure with the spots and surfaces represents SynB3‐PVGLIG‐PTX. B) The graph of partial enlargement shows the binding site of MMP‐2 (ribbon and sphere) and SynB3‐PVGLIG‐PTX. The cyan ribbon and the blue spheres represent MMP‐2 and its hydrophilic binding region, respectively. In the structure of SynB3‐PVGLIG‐PTX, the carbon (C), oxygen (O), nitrogen (N) and hydrogen (H) are represented by dark‐gray, red, blue, and light‐gray balls, respectively. C) The 3D structure between SynB3‐PVGLIG‐PTX and corresponding amino residue of MMP‐2 is shown. Gray dashed lines represent the hydrogen bond interactions between SynB3‐PVGLIG‐PTX and MMP‐2. D) The interaction mode of SynB3‐PVGLIG‐PTX with MMP‐2 is presented, where the hydrophilic and hydrophobic amino acid residues are labeled.

MMP‐2 is vital to the process of extracellular matrix breakdown through the recognition and cleavage of a special sequence of the peptide. Moreover, as reported previously, MMP‐2 is over‐expressed in GBM cells to directly promote cell invasion and migration.^[^ ^34^ ^]^ For this reason, several PDCs were designed and developed based on the CPPs and the MMP‐2‐sensitive linker (PVGLIG). Molecular docking between PDC and MMP‐2 was examined using the Discovery Studio software. The results of the docking scores of different conjugates on MMP‐2 are listed in **Table** **1**. In particular, the lowest binding free energy occurred between SynB3‐PVGLIG‐PTX (C4) and MMP‐2 (−151.29 kcal mol^−1^), revealing the best binding affinity on MMP‐2 compared with nine other conjugates. The predicted binding mode between SynB3‐PVGLIG‐PTX and MMP‐2 is shown in **Figure** **1**. SynB3‐PVGLIG‐PTX forms hydrogen bonds with the residues ASP46, LEU89, GLU95, ARG98, GLY216, GLU217, SER369, GLY371, LYS372, GLN393, LYS429, and GLU484. Furthermore, it interacts with the residues ARG98, GLY218, GLN219, VAL220, and TYR232 through the conjugate force. Moreover, this conjugate demonstrated a hydrophobic interaction with the LEU548 residue, likely promoting binding stability between MMP‐2 and SynB3‐PVGLIG‐PTX. Accordingly, the SynB3‐PVGLIG‐PTX with the lowest docking score was further studied.

**Table 1:** PDCs and their docking score with MMP‐2

ID	PDCs	Docking score [kcal mol^−1^]**^e)^**
C1	**PTX**‐**2′OH**‐malonyl‐PVGLIG‐pVEC ^a)^, ^b)^, ^c)^, ^d)^	−59.94
C2	**PTX**‐**2′OH**‐malonyl‐PVGLIG‐TP10	−65.13
C3	**PTX**‐**2′OH**‐malonyl‐PVGLIG‐TP10‐2	−83.17
C4	**PTX**‐**2′OH**‐malonyl‐PVGLIG‐SynB3	−151.29
C5	**PTX**‐**2′OH**‐malonyl‐PVGLIG‐Tat 47–57	−32.85
N1	pVEC‐PVGLIG‐**2′OH**‐**PTX**	−44.71
N2	TP10‐PVGLIG‐**2′OH**‐**PTX**	−110.29
N3	TP10‐2‐PVGLIG‐**2′OH**‐**PTX**	−110.53
N4	SynB3‐PVGLIG‐**2′OH**‐**PTX**	−19.20
N5	Tat 47‐57‐PVGLIG‐**2′OH**‐**PTX**	−33.25

^a)^ The nomenclature of PTX‐2′OH refers to that 2′‐OH group of PTX that was used to react with the peptide for forming the PDCs;

^b)^ Underlined residues are the sequence of the MMP‐2‐sensitive peptide;

^c)^ The group indicated in red was the linker between PTX and peptide;

^d)^ The green moiety were the CPPs;

^e)^ The binding free energy between the conjugate and MMP‐2 was represented in the form of kcal mol^−1^ as the docking score.

### 2.2 Nanoparticle Tracking Analysis (NTA)

**Figure 2:**
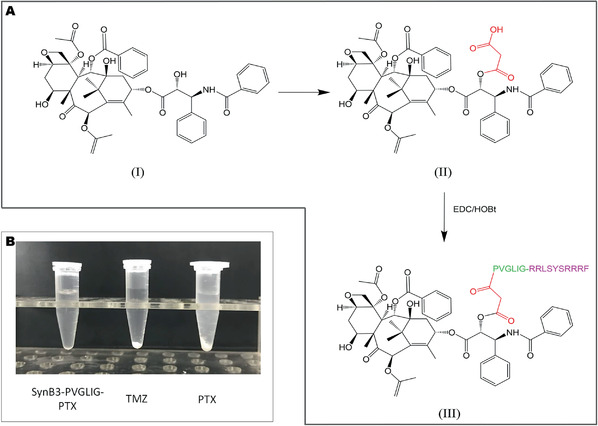
A) Synthesis procedure of SynB3‐PVGLIG‐PTX and B) the solubility of SynB3‐PVGLIG‐PTX, TMZ, and PTX at the concentration of 3 mg mL^−1^ in PBS.

**Figure 3:**
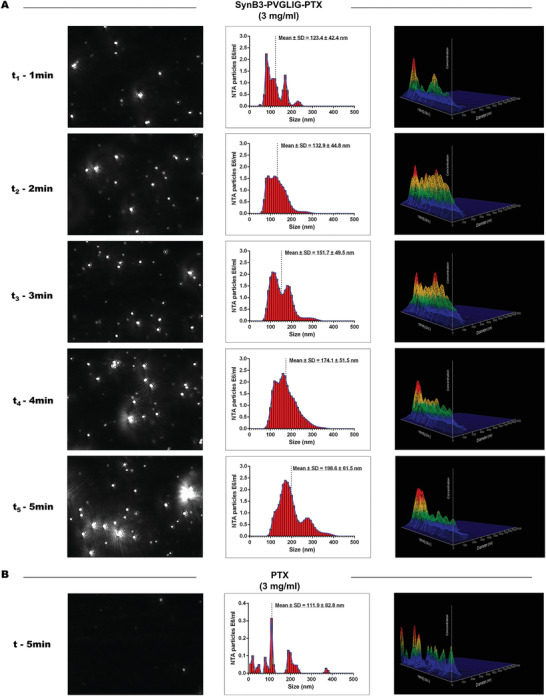
Real‐time size distribution of SynB3‐PVGLIG‐PTX (3 mg mL^−1^) and PTX (3 mg mL^−1^) measured by NTA. The size distribution of particles measured by NTA (the middle panels) with the corresponding screenshot from the video captured by the system of NTA (the left panels), and 3D graph (diameter vs intensity vs concentration; the right panels) are presented. A) The solution of SynB3‐PVGLIG‐PTX (3 mg mL^−1^) was measured by NTA every minute after preparation. B) The solution of PTX at the concentration of 3 mg mL^−1^ was measured by NTA in 5 min after preparation.

After the synthetic procedure described in **Figure** **2**A was performed, SynB3‐PVGLIG‐PTX was produced and dissolved in PBS. As expected, the solubility of SynB3‐PVGLIG‐PTX was significantly higher than those of PTX and TMZ. Figure 2B shows that the SynB3‐PVGLIG‐PTX solution (3 mg mL^−1^) was colorless and transparent, whereas PTX and TMZ were nearly insoluble in PBS at the same concentration. We considered that the solubility of SynB3‐PVGLIG‐PTX was enhanced, probably attributable to the characteristics of the peptide. In this conjugate, the dual‐functional peptide, SynB3‐PVGLIG, is hydrophilic, acting as the “shell” to cover the hydrophobic PTX and to achieve higher water solubility of the conjugate. NTA measurement was performed to investigate the behavior of conjugate aggregation. The ready‐prepared solution of SynB3‐PVGLIG‐PTX was injected into the sample chamber immediately, while the videos were recorded and analyzed (**Figure** **3**A). At t1, the mean size of particles obtained by NTA was 123.4 nm, most of which were particles with size around 82.0 nm. At 2 min (t2), the mean size of the particle increased to 132.9 nm. Subsequently, particles with large sizes (>300.0 nm) were detected at t3. At t4, the mean size at the upper concentration was 174.1 nm, significantly larger than that at t1. At the end (t5), almost all the particles had sizes above 100.0 nm, and the mean particle size reached 198.6 nm. In contrast, the mean size of PTX in PBS was maintained at around 111.9 nm within 5 min (Figure 3B). Together, these results demonstrated that SynB3‐PVGLIG‐PTX could aggregate in the PBS, and that the particle size grew over time, which partially indicated that the SynB3‐PVGLIG‐PTX could aggregate to enhance water solubility.

### 2.3 Characterization of the SynB3‐PVGLIG‐PTX Nanocomplex

**Figure 4:**
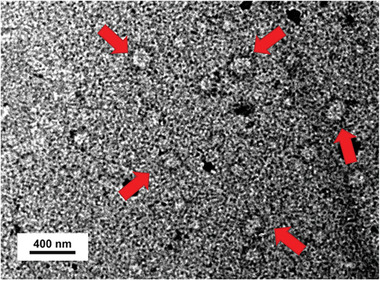
Characterizations of SynB3‐PVGLIG‐PTX (3 mg mL^−1^) via TEM. TEM images of the SynB3‐PVGLIG‐PTX nanocomplex stained with phosphotungstic acid are shown. The red arrows indicate the representative nanocomplex where steady state is considered to have been reached.

In order to assess the characteristics of the nanocomplex, the size and zeta potential (ZP) of SynB3‐PVGLIG‐PTX were determined through Dynamic Light Scattering (DLS) and a ZP analyzer. Results are shown in **Table** **2**. It was apparent that the nanocomplex dispersed as the individual nanoparticles were homogeneously distributed with an extremely low polydispersity index (PDI; 0.041–0.116). The mean diameter of the nanocomplex was 161.7 nm, which is very close to the average diameter obtained in the NTA (Table 2). Notably, the results of ZP showed that the nanocomplex carried a strong positive charge (20.45–25.94 mV) on its surface (Table 2). Beyond that, the morphologies of the nanocomplex were observed via transmission electron microscope (TEM), which displayed that the nanocomplex had a well‐defined spherical shape (**Figure** **4**). These results confirmed that SynB3‐PVGLIG‐PTX could indeed polymerize to form a positively charged stabilized nanocomplex, in which the positively charged amino acid residues played an important role in this nano structure.

**Table 2:** Characterization of the SynB3‐PVGLIG‐PTX nanocomplex

PDC	Mean Size [nm]^a)^	Size [nm]^b)^	PDI^b)^	ZP [mV]^c)^
SynB3‐PVGLIG‐PTX	162.7	150.0	0.116	25.94
		178.6	0.041	20.45
		156.4	0.090	22.08

^a)^ The mean size of nanocomplex obtained via the NTA;

^b)^ Determined with a DLS in PBS at 3 mg mL^−1^;

^c)^ Determined with a ZP analyzer in PBS at 3 mg mL^−1^.

### 2.4 The Cleavability of SynB3‐PVGLIG‐PTX by MMP‐2

To study the hydrolysis ability of MMP‐2 to SynB3‐PVGLIG‐PTX, the contents of SynB3‐PVGLIG‐PTX and PVG‐PTX in the hydrolysate were ascertained using HPLC/MS (Figure S1, Supporting Information). The percentage of cleaved SynB3‐PVGLIG‐PTX is profiled in Figure S1E (Supporting Information; blue line and square). As expected, PVG‐PTX could be detected after enzymatic hydrolysis with MMP‐2 for 0.5 h, after which about 41.18% SynB3‐PVGLIG‐PTX was cleaved by MMP‐2. Over time, the percentage of PVG‐PTX released from SynB3‐PVGLIG‐PTX increased rapidly and reached 90.91% after incubation with MMP‐2 for 1 h. After incubation with MMP‐2 for about two hours, SynB3‐PVGLIG‐PTX completely disappeared, while the percentage of PVG‐PTX peaked (100%). In contrast, SynB3‐PVGLIG‐PTX could not be cleaved without MMP‐2, and neither PVG‐PTX nor free PTX could be detected (Figure S1F, Supporting Information; red line and triangle). Moreover, SynB3‐PTX, which does not contain a cleavable peptide linker, could not be cleaved even in the presence of MMP‐2, and neither free PTX nor fragments were detected (Figure S1G, Supporting Information; black line and square). These results are consistent with our previous expectations that the release of SynB3‐PVGLIG‐PTX depends on the MMP‐2‐mediated cleavage of the sensitive linker (PVGLIG), and that both are indispensable.

### 2.5 Inhibition of MMP‐2 Expression by siRNA and Western Blot Analysis

**Figure 5:**
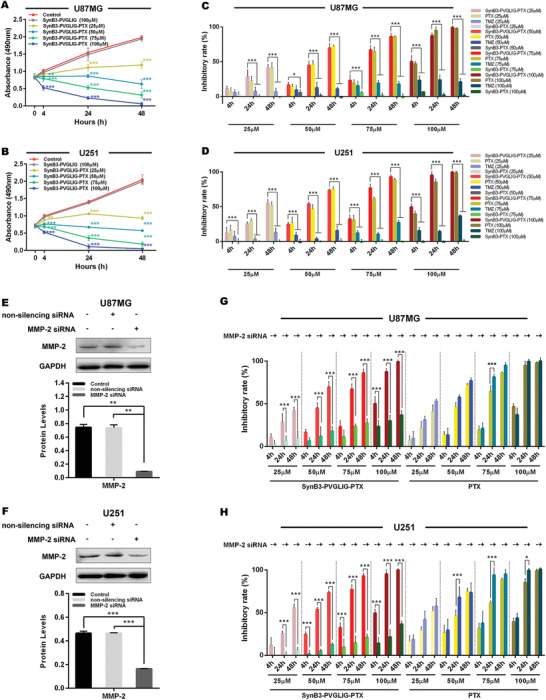
The effect of SynB3‐PVGLIG‐PTX on cell proliferation in different GBM cells using the MTS assay. All data in this assay are represented as mean ± SD of three independently repeated experiments. A,B) The growth curves of U87MG and U251 cell lines treated with various concentration of SynB3‐PVGLIG‐PTX (25 × 10^−6^ to 100 × 10^−6^ м) for 4, 24, and 48 h, as indicated. SynB3‐PVGLIG‐PTX notably reduced proliferation of GBM cells (U87MG and U251) when compared with the control group (data presented as mean ± SD, *n* = 5, *p*‐values are calculated using two‐way ANOVA with Dunnett's post‐hoc test, **p < 0.01, ***p < 0.001). However, there was no significant difference between the dual‐functional peptide (SynB3‐PVGLIG) group and control group. C,D) The cellular proliferative inhibitory rates of SynB3‐PVGLIG‐PTX, PTX, TMZ, and SynB3‐PTX at different incubating concentrations (25 × 10^−6^, 50 × 10^−6^, 75 × 10^−6^, and 100 × 10^−6^ м) and time (4, 24, and 48 h) are presented as histograms. Data presented as mean ± SD, *n* = 5, *p*‐values are calculated using two‐way ANOVA with Tukey's post‐hoc, *p < 0.05, ***p < 0.001. E,F) MMP‐2 expression was efficiently down‐regulated by transfecting MMP‐2 siRNA in U87MG and U251, as determined by western blots. GAPDH was classified as the loading control. Data presented as mean ± SD, *n* = 5, *p*‐values are calculated using one‐way ANOVA with Tukey's post‐hoc, **p < 0.01; ***p < 0.001. G,H) The inhibitory effects of SynB3‐PVGLIG‐PTX and PTX on the proliferation of U87MG, MMP‐2 siRNA‐transfected U87MG, U251, and MMP‐2 siRNA‐transfected U251 were shown in accordance with different treated times (4, 24, and 48 h) and concentrations (25 × 10^−6^, 50 × 10^−6^, 75 × 10^−6^, and 100 × 10^−6^ м). Data presented as mean ± SD, *n* = 5, *p*‐values are calculated using two‐way ANOVA with Tukey's post‐hoc, *p < 0.05, ***p < 0.001.

To detect the antiproliferative effect of SynB3‐PVGLIG‐PTX with and without MMP‐2 in GBM cell lines, the MMP‐2 siRNA was used for establishing the U87MG and U251 cell lines with MMP‐2 down‐regulated expression. The western blot assay was performed to detect variations in MMP‐2 protein levels (48 h post‐transfection). The results of the western blot revealed that MMP‐2 expression levels in the MMP‐2 siRNA group was significantly decreased compared with that in the control group (**p < 0.01, ***p < 0.001, **Figure** **5**E,F). There was no significant difference between the control group and the nonsilencing siRNA group in the expression level of MMP‐2, suggesting that MMP‐2 siRNA inhibited MMP‐2 expression. In a follow‐up MTS assay and Transwell experiment, the untreated control cells and MMP‐2 siRNA cells were taken as the experimental objects to assess the effect of MMP‐2 on the inhibitory rate of different drugs in GBM cell lines. All the transfected cells used in the MTS assay and Transwell assay were selected after 24 h transfection by MMP‐2 siRNA.

### 2.6 Time‐ and Concentration‐dependent Effect of SynB3‐PVGLIG‐PTX on Cell Proliferation with and Without MMP‐2

To assess the antiproliferative effect of SynB3‐PVGLIG‐PTX on MMP‐2 positive and negative GBM cell lines, a cell proliferation assay was performed based on MTS. U87MG, U251, MMP‐2 siRNA‐transfected U87MG, and MMP‐2 siRNA‐transfected U251 were treated with SynB3‐PVGLIG‐PTX at different concentrations (25 × 10^−6^, 50 × 10^−6^, 75 × 10^−6^, and 100× 10^−6^ м) for 4 h, 24 h and 48 h, respectively. These cells were treated with PTX and TMZ at different concentrations (25 × 10^−6^, 50 × 10^−6^, 75 × 10^−6^, and 100 × 10^−6^ м) as the drug control, and SynB3‐PVGLIG as the dual‐functional peptide control. The cells with the highest absorbance indicate that most of the cells were alive. The absorbance of live cells in the SynB3‐PVGLIG‐PTX group decreased with a rise in the SynB3‐PVGLIG‐PTX concentration and the prolonging of its exposure time, suggesting that SynB3‐PVGLIG‐PTX inhibited the proliferation of GBM cells (U87MG and U251) in a time‐ and dose‐dependent manner as compared with the control groups (**p < 0.01, ***p < 0.001, Figure 5A,B). As shown in Figure 5C,D, the proliferative inhibitory rate of SynB3‐PVGLIG‐PTX on U87MG and U251 increased over time and with increasing concentrations, and were significantly higher than those in the TMZ groups (***p < 0.001). There was no significant difference in the cell proliferative inhibitory rate between the SynB3‐PVGLIG‐PTX group and the PTX group (*p* > 0.05). Overall, the half maximal inhibitory concentrations (IC50s) of SynB3‐PVGLIG‐PTX against U87MG and U251 were 45.787 × 10^−6^ and 41.413 × 10^−6^ м, respectively, when calculated at 24 h (**Table** **3**). After 48 h treatment, the IC50 of SynB3‐PVGLIG‐PTX was 31.981 × 10^−6^ м in U87MG and 27.366 × 10^−6^ м in U251, only taking up one sixteenth and one seventh of the IC50 of TMZ, respectively (Table 3). These results demonstrated that SynB3‐PVGLIG‐PTX could inhibit the proliferation of GBM cells significantly.

**Table 3:** IC50 values of TMZ, PTX, and SynB3‐PVGLIG‐PTX toward U87MG, U251, and their MMP‐2 siRNA‐transfected cell lines

Group	IC50 [× 10^−6^ м]^a)^											
	U87MG			U87MG+MMP‐2 siRNA			U251			U251+MMP‐2 siRNA		
	4 h	24 h	48 h	4 h	24 h	48 h	4 h	24 h	48 h	4 h	24 h	48 h
TMZ	392.123	382.285	514.467	470.585	413.904	280.138	3072.756	660.490	212.816	3298.487	869.053	130.888
PTX	148.470	46.321	31.285	208.942	37.258	28.519	180.545	47.130	27.350	135.507	31.542	23.624
SynB3‐PVGLIG‐PTX	139.541	45.787	31.981	311.219	219.364	172.180	115.846	41.413	27.366	165.602	207.674	176.806

^a)^ IC50 values denote the concentrations of different drugs necessary for 50% inhibition of cell proliferation activity. The periods of drug treatment were 4, 24, and 48 h.

However, in the MMP‐2 siRNA‐transfected U87MG and U251, SynB3‐PVGLIG‐PTX could result in an extremely lower inhibitory effect on cell proliferation than that treated at the same concentration ranges (25 × 10^−6^, 50 × 10^−6^, 75 × 10^−6^, and 100 × 10^−6^ м) in the U87MG and U251 cell lines (Figure 5G,H). The absence of MMP‐2 manifested as an obviously reducing trend of IC50 of SynB3‐PVGLIG‐PTX in both cell lines (Table 3). These results indicated that SynB3‐PVGLIG‐PTX could only inhibit cell proliferation based on the presence of MMP‐2, which suggested that SynB3‐PVGLIG‐PTX performs a specific inhibitory action on the proliferation of GBM cells.

In addition, in order to further verify the mechanism underlying the specific inhibitory activity of SynB3‐PVGLIG‐PTX on GBM cell proliferation, a supplementary experiment was performed using SynB3‐PTX‐treated cells as inactive control groups. The results showed that the inhibition rates of SynB3‐PTX without an MMP‐2‐sensitive linker (PVGLIG) in U87MG and U251 were extremely low, at a near‐zero level (Figure 5C,D). Based on these observations, we confirmed that SynB3‐PVGLIG‐PTX has a specific antigrowth activity, that is, only in the presence of MMP‐2, the sensitive linker (PVGLIG) contained in SynB3‐PVGLIG‐PTX can be specifically hydrolyzed to release PTX, thereby inhibiting the proliferation of GBM cells.

### 2.7 SynB3‐PVGLIG‐PTX Inhibits Migration and Invasion in the Presence of MMP‐2 In Vitro

To evaluate the effect of MMP‐2 on SynB3‐PVGLIG‐PTX for inhibiting migration and invasion in different cell lines (U87MG, U251, and their corresponding siRNA transfected cell lines), the Transwell assay was performed. As shown in Figures S2 and S3 (Supporting Information), SynB3‐PVGLIG‐PTX (50 × 10^−6^ м) could statistically significantly decrease the migratory and invasive activity of GBM cells (U87MG and U251) compared with the control group (***p < 0.001), TMZ group (50 × 10^−6^ м; ***p < 0.001) and SynB3‐PVGLIG group (50 × 10^−6^ м; ***p < 0.001), while the relative ratios of migration and invasion between the 50 × 10^−6^ м SynB3‐PVGLIG‐PTX group and 50 × 10^−6^ м PTX group were not statistically different (*p* > 0.05). MMP‐2, as a member of matrix metalloproteinases (MMPs), is vital to the migration and invasion of tumor cells that due to MMP‐2 can degrade the main constituent of membranes (type IV collagen). Thus, the siRNA transfected cells displayed slower migration and invasion compared with the nontransfected cell lines (Figures S2 and S3, Supporting Information). Interestingly, as shown in Figures S2 and S3 (Supporting Information), the MMP‐2 siRNA transfected cells in the SynB3‐PVGLIG‐PTX group exhibited significantly higher migratory and invasive activity compared with nontransfected cells treated with SynB3‐PVGLIG‐PTX (***p < 0.001). In brief, these results revealed that SynB3‐PVGLIG‐PTX could effectively inhibit the migration and invasion of GBM cells in the presence of MMP‐2 in vitro, and that SynB3‐PVGLIG‐PTX does not exhibit independent antimigratory and anti‐invasive actions without being assisted by the proteolytic effect of MMP‐2.

### 2.8 The antitumor Efficacy of SynB3‐PVGLIG‐PTX In Vivo

**Figure 6:**
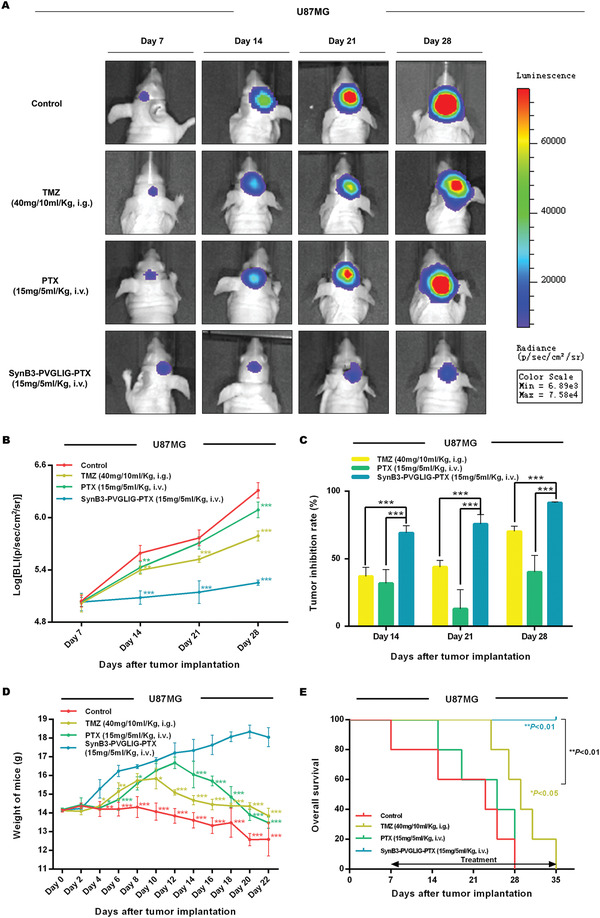
Effect of SynB3‐PVGLIG‐PTX on tumor growth, body weight, and overall survival of U87MG bearing nude mice. A) In vivo bioluminescence images of the intracranial glioma xenograft nude mice generated with the indicated U87MG cells after administration of TMZ (40 mg/10 mL kg^−1^, i.g., five times a week), PTX (15 mg/5 mL kg^−1^, i.v., twice a week), or SynB3‐PVGLIG‐PTX (15 mg/5 mL kg^−1^, i.v., twice a week). Representative images of mice are shown. B) Fluorescence intensity was recorded as p s^−1^ cm^−2^ sr^−1^. The bioluminescence quantification results at days 7, 14, 21, and 28 after implantation (*n* = 5 for each group) are shown. Compared with the control group, data are expressed as mean ± SD. **p < 0.01; ***p < 0.001 by two‐way ANOVA with Tukey's post‐hoc. C) All data are expressed as mean ± SD, *n* = 5. Compared with the SynB3‐PVGLIG‐PTX group, ***p < 0.001 by two‐way ANOVA with Tukey's post‐hoc. D) The weights of mice were measured every other day, which are expressed as mean ± SD, *n* = 5. Compared with the SynB3‐PVGLIG‐PTX group, *p < 0.05, **p < 0.01, ***p < 0.001 by two‐way ANOVA with Dunnett's post‐hoc. E) Kaplan–Meier analysis of the overall survival of glioma‐bearing nude mice. *p < 0.05 for the difference between the control group and the TMZ group, **p < 0.01 for TMZ group versus SynB3‐PVGLIG‐PTX group, **p < 0.01 for control group versus SynB3‐PVGLIG‐PTX group.

The antitumor effect of SynB3‐PVGLIG‐PTX was subsequently evaluated in vivo. After 7 days of orthotopic implantation, the total photon count in U87MG tumor‐bearing mice was around 1.09 × 10^5^±0.18 × 10^5^ photons per second per centimeter square per steradian (p s^−1^ cm^−2^ sr^−1^, **Figure** **6**A), suggesting that all mice models were successfully established and could be used for further antitumor efficacy assays. These mice were randomly split into four groups and treated using the method described previously. The representative cerebral bioluminescence images of mice in each group taken weekly are shown in Figure 6A. After the initiation of the treatment (at day 7), the bioluminescence signals of tumors in all groups increased by a certain extent. In particularly, the log of bioluminescence radiance (BLI) in the SynB3‐PVGLIG‐PTX group was significantly lower than that in the control group by day 14 (***p < 0.001, Figure 6B), while after the administration of SynB3‐PVGLIG‐PTX, there were few differences in the tumor signals between day 7 and day 14 (Figure 6A). At all later days, the differences in log (BLI) between the SynB3‐PVGLIG‐PTX and control group were significantly increased (***p < 0.001, Figure 6B), in which the mouse treated with SynB3‐PVGLIG‐PTX yielded a notably weaker bioluminescence radiance and a concurrently smaller bioluminescent area than those in the control group. On day 28, mice treated with SynB3‐PVGLIG‐PTX achieved the highest tumor inhibition rate (91.40±0.57%), which was 2.27‐fold and 1.30‐fold higher than that of the PTX and TMZ groups, respectively (***p < 0.001, Figure 6C). From the results of the bioluminescence assays, we concluded that SynB3‐PVGLIG‐PTX could sufficiently suppress the growth of intracerebral tumors in vivo after 28 days compared with the inhibition rate yielded by TMZ or PTX (***p < 0.001, Figure 6C). In addition, the weight and overall survival period of mice administered with SynB3‐PVGLIG‐PTX (15 mg/5 mL kg^−1^, i.v.) were significantly higher than those in the control group (*p < 0.05, **p < 0.01, ***p < 0.001, Figure 6D,E). Again, the separation in survival curves was notable among the four groups, with survival medians equal to 23, 29, and 25 for the control group, TMZ group and PTX group, respectively. In contrast, there was no death in the SynB3‐PVGLIG‐PTX group across the assays. Especially concerning was that, the administration concentration of SynB3‐PVGLIG‐PTX was 15 mg/5 mL kg^−1^, which is equivalent to 5.25 µmol/5 mL kg^−1^. Theoretically, after SynB3‐PVGLIG‐PTX at this concentration is completely hydrolyzed by MMP‐2, the concentration of free PTX released is 4.49 mg/5 mL kg^−1^, which is only one‐third of the concentration administered in the PTX group. The experimental results show that, compared with the group given a high dose of PTX (15 mg/5 mL kg^−1^), the tumor volume of mice given the nanoconjugate (SynB3‐PVGLIG‐PTX) was significantly reduced, and its overall survival was also significantly prolonged, which indicates that SynB3‐PVGLIG‐PTX has a significant antiglioma activity in vivo. Accordingly, SynB3‐PVGLIG‐PTX, as a PDC that combined PTX to a dual‐functional peptide consisting of SynB3 and a MMP‐2‐sensitive peptide, could observably improve survival and decrease weight loss over the PTX monotherapy in vivo, and significantly overmatched the mice treated with TMZ.

**Figure 7:**
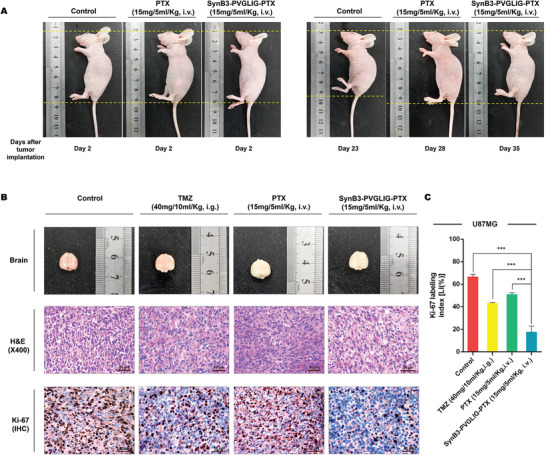
Proliferative indices of U87MG xenograft tumors after administration. A) Representative images of nude mice at day 2 and the day of death after tumor implantation in the control group, PTX group, and SynB3‐PVGLIG‐PTX group. From these images, it was found that the height of mice treated with PBS as the control was significantly decreased, while there was no substantial change in PTX group and SynB3‐PVGLIG‐PTX group. This result is consistent with body weight results, further indicating that PTX and SynB3‐PVGLIG‐PTX could facilitate survival in mice. B) Macroscopic appearance, H&E staining images of tumors, and IHC of Ki‐67 in outgrowing tumor slices. Scale bars for H&E and IHC: 50 µm (× 400). All images of representative tumors are presented. C) Comparisons of LIs (%) of Ki‐67 in the xenograft tumor tissue from different groups. All experiments were performed at least in triplicate and the data are denoted as mean ± SD, *n* = 5. ***p < 0.001 compared with the SynB3‐PVGLIG‐PTX group by one‐way ANOVA with Tukey's post‐hoc.

To further verify the suppressive effect of SynB3‐PVGLIG‐PTX on the proliferation of tumor cells in vivo, all the mice were sacrificed and brain tissue containing tumors were excised (**Figure** **7**A,B). The results of hematoxylin and eosin (H&E) and immunohistochemistry (IHC) strongly suggested that the mouse xenograft model treated with SynB3‐PVGLIG‐PTX exhibited a significantly lower growth rate and cell density compared with the control group, TMZ group, and PTX group (***p < 0.001, Figure 7B,C). Furthermore, the Ki‐67 index in the SynB3‐PVGLIG‐PTX group was 17.77%, which was about 1/4, 2/5, and 1/3 of that in the control, TMZ, and PTX groups, respectively. Accordingly, these results validated the notion that SynB3‐PVGLIG‐PTX could efficiently suppress the growth of GBM cells in vivo, which is consistent with the results in vitro. Overall, these results implied that SynB3‐PVGLIG‐PTX, as a typical PDC, could act as a potentially novel delivery nanocomplex for the treatment of GBM.

### 2.9 The Biodistribution Analysis and BBB Penetration Study of SynB3‐PVGLIG‐PTX In Vivo

**Figure 8:**
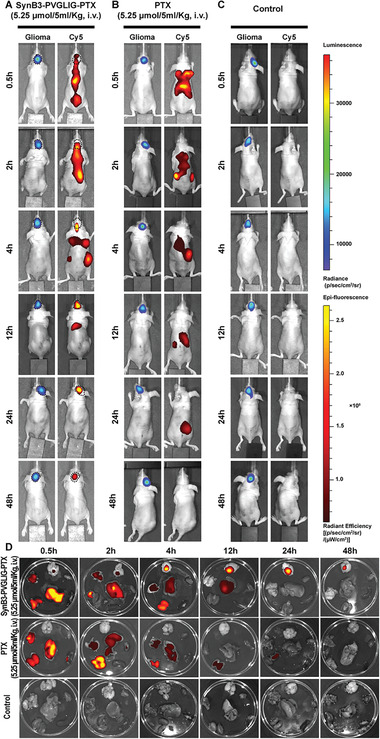
Biodistribution of SynB3‐PVGLIG‐PTX in U87MG xenograft nude mice. U87MG cells transfected with a luciferase reporter were implanted into the right cerebral hemisphere of 6‐week‐old male nude mice. Tumor size and location were determined by bioluminescent imaging using the IVIS Lumina Imaging System. On the 14th day after transplantation, the radiance of tumors was about 2.0 × 10^5^ p s^−1^ cm^−2^ sr^−1^, that is, all mice had been successfully modeled. U87MG xenograft nude mice were then treated with the indicated drug dose, and real‐time fluorescence images of tumor and drug distribution were taken at 0.5, 2, 4, 12, 24, and 48 h. A) Representative images of mice administered with SynB3‐PVGLIG‐PTX. The outline and location of the orthotopic xenograft tumor in the mouse brain is outlined with a black dotted line. (B) and (C) are representative images of the PTX group and the control group, respectively. D) Bioluminescent images of different organs. Top: brain; upper left: lungs; bottom left: kidneys; upper right: heart; bottom right: spleen; center: liver.

Given the above results, it was suspected that the SynB3‐PVGLIG‐PTX generated better antitumor activity than free PTX in the xenograft nude mice due to the intervention of the dual‐functional peptide (SynB3‐PVGLIG), which made it easier for SynB3‐PVGLIG‐PTX to enter the brain and release the loading‐PTX at the tumor site. Thus, a biodistribution analysis and BBB penetration study of SynB3‐PVGLIG‐PTX in vivo was performed. In this study, the fluorescence image of nude mice was captured and analyzed. As shown in **Figure** **8**A, after the injection of SynB3‐PVGLIG‐PTX through the tail vein, the fluorescence signal (Cy5) of SynB3‐PVGLIG‐PTX was mainly detected in the bladder, liver, kidneys, lungs, and brain. In detail, at 0.5 h after the injection of SynB3‐PVGLIG‐PTX, the fluorescent signal could be detected at the tumor site in the brain. With the passage of time, the fluorescent signal in the brain tumor gradually increased and peaked at 12 h. At 24 h, the fluorescent signal could not be detected in any other tissue except the brain. At the end of the experiment (48 h), the fluorescent signal could still be detected in brain. At each time point, the mice were sacrificed, and their major organs (e.g., brain, heart, lungs, liver, spleen, and kidneys) were collected for further fluorescence detection in vitro. The results in vitro (Figure 8D) were highly consistent with the real‐time fluorescence imaging results in vivo. In addition, after locating the size and distribution of brain tumors in the xenograft nude mice (marked by the thick black dots in Figure 8A), we found that the fluorescent signal of the targeted drug was mainly distributed in the transplanted gliomas, but the signal was very limited in nontumor brain tissue. These experimental results indicate to a certain extent that SynB3‐PVGLIG‐PTX can not only cross the BBB, but that it also has a certain specificity, which caused it to mainly collect in glioma tissue.

Compared with mice administered with SynB3‐PVGLIG‐PTX, the experimental results showed that PTX was mainly distributed in the lungs, liver, and kidneys, and that no drug distribution was detected in the tumor site in the brain (Figure 8B). With time, PTX was mainly found in the liver and kidneys (4 h). From 12 h to 24 h, the drug was only distributed in the kidneys after which no signal was detected in vivo at 48 h. Furthermore, we did not find a detectable fluorescence signal (Cy5) in the control group (Figure 8C). The fluorescence images of the organs from these mice were also consistent with the real‐time distribution results in vivo (Figure 8D).

### 2.10 Study on the Ability of SynB3‐PVGLIG‐PTX to Release PTX in the Mouse Brain

**Figure 9:**
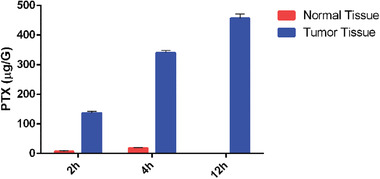
The concentration‐time profiles of PTX in different brain tissues. This experiment was performed following a single dose administration of SynB3‐PVGLIG‐PTX (5.25 µmol/5 mL kg^−1^, i.v.) in U87MG xenograft nude mice. Data presented as mean ± SD, *n* = 5.

In this study, the nude mice were sacrificed, and the concentration of free PTX both in the brains without the tumor and those with the tumor was ascertained using the HPLC/MS method. The concentration‐time column of PTX in different cerebral tissue of nude mice with orthotopic implantation after the caudal vein injection of SynB3‐PVGLIG‐PTX is plotted in **Figure** **9**. As expected, intravenous injection of SynB3‐PVGLIG‐PTX caused the PTX concentration in the tumor to be significantly higher than that in normal brain tissue. Therein, the concentration of PTX in the tumor tissue reached 136.76 µg G^−1^ at 2 h, which was 20 times that of the normal brain. Subsequently, increasingly higher levels of PTX were detected in the tumor tissue of mice until the concentration of PTX finally reached 456.32 µg G^−1^. On the other hand, in the normal brain tissue, the PTX concentration remained essentially constant and relatively low, ranging from about 0.23 to 18.68 µg G^−1^. These results confirmed our supposition that SynB3‐PVGLIG‐PTX has a better BBB penetrability and is mainly transported into brain tumor tissue, which could lead to a higher cytotoxicity in vivo resulting in high anti‐GBM activity.

## 3 Discussion

This study was aimed at enhancing BBB penetrability and specificity of PTX by employing a strategy of PDCs assisted by a dual‐functional peptide. In recent years, numerous insightful studies have demonstrated that MMP‐2 is over‐expressed in GBM and has a characteristic ability to recognize and cleave peptides with specific sequences.^[^ ^28^, ^29^, ^35^ ^]^ As such, various substrates of MMP‐2 were designed to enhance specificity for drug delivery, thus leading to a remarkable effort toward the investigation of drug‐targeted delivery systems. The present study aimed to conjugate PTX with the effective MMP‐2‐sensitive linker, PVGLIG to reduce off‐target binding and side effects.^[^ ^36^, ^37^ ^]^ However, to treat intracranial tumors, enhancement of BBB permeability of a drug was the cardinal problem that needed to be solved. As BBB‐penetrating moieties that have been extensively studied, CPPs (e.g., SynB3) were used to complement our dual‐functional peptide for efficiently treating GBM in this study. Accordingly, multiple PDCs were designed and evaluated by computer simulation. The results demonstrated that SynB3‐PVGLIG‐PTX had the lowest binding free energy with MMP‐2, revealing that SynB3‐PVGLIG‐PTX could be specifically recognized and cleaved by MMP‐2 and also suggesting its potential value as a drug delivery system for GBM therapy.

PTX is poorly soluble in water, which has led to many side effects in the clinic. In contrast, the defect of PTX could be rescued by the dual‐functional peptide. In our study, SynB3‐PVGLIG‐PTX could be readily dissolved in PBS. By studying the structural features of SynB3‐PVGLIG‐PTX, it was found that a portion of the dual‐functional peptide (SynB3‐PVGLIG) was hydrophilic, which likely moved outward and could be connected under the action of hydrogen bonding between amino acid residues such as arginine (R), serine (S), and tyrosine (Y), which ultimately resulted in enwrapping the PTX. The formation of this structure was probably responsible for the solubility improvement of SynB3‐PVGLIG‐PTX. To support this notion, the NTA assay was performed. The images showed that SynB3‐PVGLIG‐PTX could aggregate, in which the size of particles increased over time. In contrast, the size of PTX did not vary for 5 min and held steady at 111.9 ± 82.8 nm. These findings proved to a certain extent that SynB3‐PVGLIG‐PTX is a drug‐peptide nanocomplex that can assemble to form a special structure with good solubility in water. In order to further elaborate its structural characteristics, the PDI and TEM analyses were performed, and we found that PDC can indeed form a nanoconjugate with a uniform size (about 162 nm) in PBS. Remarkably, this nanoconjugate has a strong positive charge, which is mainly due to the fact that the SynB3 portion of the nanoconjugate exposed to the outside contains a large number of positively charged arginine (R) residues. These results further confirm our hypothesis that SynB3‐PVGLIG‐PTX can form a hydrophilic, uniform, and positively charged nanoconjugate in PBS, which greatly increases the water solubility of PTX. In the following release study, we found that SynB3‐PVGLIG‐PTX could be recognized and hydrolyzed by MMP‐2, while the hydrolysates could not be detected in the absence of MMP‐2 or with a lack of the MMP‐2‐sensitive linker (PVGLIG). These results strongly indicated that the SynB3‐PVGLIG‐PTX nanoconjugate was soluble in water, which could release the loading drug by MMP‐2 specific hydrolysis.

It is known that MMP‐2 is over‐expressed in glioma tissue, while there is almost no expression in normal brain tissue.^[^ ^30^, ^31^ ^]^ Unlike with most chemotherapeutics, we could eliminate the release of the nanopreparation in nontumor cells without MMP‐2. In the following antitumor investigations in vitro, the U87MG and U251 cell lines acted as a model of GBM in vitro and those transfected with MMP‐2 siRNA as the control (MMP‐2‐total knockdown). Using MTS and Transwell assays, it was found that SynB3‐PVGLIG‐PTX could efficiently suppress the proliferation, migration, and invasion of GBM cells in vitro, which is superior to TMZ, PTX and SynB3‐PVGLIG. Notably, however, antitumor efficacies of SynB3‐PVGLIG‐PTX depended on the presence of MMP‐2, which could not inhibit the proliferation, migration, and invasion in the MMP‐2 siRNA‐transfected U87MG and U251 cell lines. Accordingly, during this antitumor process, the MMP‐2 was necessarily required, since the corresponding release of PTX from SynB3‐PVGLIG‐PTX was a highly targeting course. In addition, we also used SynB3‐PTX as an inactive control drug to conduct a supplementary experiment to improve our results. We found that PDC without the MMP‐2‐sensitive linker (PVGLIG) could not achieve the tumor suppressive activity of SynB3‐PVGLIG‐PTX in the GBM cells. Thus, the MMP‐2‐sensitive linker is a structural component necessary for the targeted release of PTX by SynB3‐PVGLIG‐PTX. These results strongly suggest that SynB3‐PVGLIG‐PTX might act as a new drug‐loaded delivery system for effectively suppressing the proliferation, migration, and invasion of GBM cells in vitro due to its structure supporting high solubility and specificity.

BBB is a significant problem in glioma treatment, likely to restrict drugs from entering the brain. To address this critical issue, an antitumor efficacy study on SynB3‐PVGLIG‐PTX in vivo was conducted, with the established U87MG tumor‐bearing mice as the study model. Our in vivo data clearly showed that SynB3‐PVGLIG‐PTX inhibited the growth of GBM successfully and efficiently. Moreover, the anti‐GBM effect of SynB3‐PVGLIG‐PTX in vivo, especially against the proliferation of GBM cells, was significantly higher than that of TMZ. The application of SynB3‐PVGLIG‐PTX could prolong the overall survival of mice and decrease the loss weight during the whole course of treatment. These results were consistent with the findings in vitro, implying that SynB3‐PVGLIG‐PTX, as a drug‐loaded nanocomplex, leads to high anticancer activity and low side effects, and demonstrates potential for the treatment of GBM in future clinical practice.

However, the application of free PTX showed a rather unsatisfactory efficacy on the inhibition of GBM in vivo, significantly weaker than that of SynB3‐PVGLIG‐PTX. The main reason for this phenomenon was thought to be that SynB3‐PVGLIG‐PTX could more freely cross the BBB and release PTX at the cranial tumor site to effectively inhibit the development of GBM in vivo, which is superior to PTX. We first analyzed the chemical structure of the SynB3‐PVGLIG‐PTX nanoconjugate to examine why it is easy to traverse the BBB. From the PDI assay, we found that SynB3‐PVGLIG‐PTX can form a water‐soluble, uniformly sized positively charged nanoconjugate. In general, mammalian cell membranes are anionic, and the membrane surface of red blood cells is negatively charged. It has been reported that vascular permeability of cationic compounds is significantly higher than that of anionic compounds, which is the result of electrostatic effects. As a clear example residue, the positively charged CPP can easily penetrate the BBB.^[^ ^44^, ^45^ ^]^ This adsorption‐mediated transport is energy‐ and concentration‐dependent, which has the advantages of low affinity and high drug loading compared with receptor‐mediated transport.^[^ ^44^, ^45^ ^]^

In this study, we applied bifunctional peptides with a certain positive charge to modify PTX to obtain SynB3‐PVGLIG‐PTX. The nanoconjugate formed by SynB3‐PVGLIG‐PTX in an aqueous solution also had a positive charge (around 22.82 mV). In order to investigate whether this positively charged nanoconjugate can be targeted into the brain through electrostatic interactions with the anions on the BBB membrane, we conducted a biodistribution analysis in vivo. The experimental results were consistent with our expectations. Compared to free PTX, which is almost undetectable in the brain, SynB3‐PVGLIG‐PTX was widely distributed in the brain, and the intensity of fluorescence signal (Cy5) increased over time. The fluorescent signal could still be detected at 48 h in the SynB3‐PVGLIG‐PTX group, whereas the signal of the PTX group had disappeared. This result further validates the view that CPP (SynB3)‐modified PTX more easily penetrates the BBB compared to free PTX, which explains why SynB3‐PVGLIG‐PTX has stronger tumor suppressive activity than free PTX in vivo.

As described above, we have verified that SynB3‐PVGLIG‐PTX can specifically release PTX only in the presence of MMP‐2 in vitro. However, its ability to release PTX in the mouse brain was not yet clear. Activated MMP‐2 is mainly located in the extracellular matrix, but there is some MMP‐2 present within cells that is activated by prooxidants to perform enzymatic hydrolysis.^[^ ^46^ ^]^ It has also been reported that MMP‐2 is localized in the cytoplasm of human glioma cells.^[^ ^47^ ^]^ Accordingly, we detected the content of released PTX in the normal brain tissue and tumor using the method of HPLC/MS after the administration of SynB3‐PVGLIG‐PTX. The experimental results revealed that the tumor content of PTX in tumor tissue increased gradually, which was significantly higher than that in normal brain tissue. The main reason for these results is that with the help of a functional peptide (SynB3‐PVGLIG), the amount of SynB3‐PVGLIG‐PTX arriving at the GBM is significantly upregulated. Some SynB3‐PVGLIG‐PTX is hydrolyzed by MMP‐2 in the extracellular matrix of glioma cells, and the released PTX plays an antitumor role in the tumor microenvironment, while other SynB3‐PVGLIG‐PTX molecules can also enter glioma cells and be hydrolyzed by MMP‐2 to release PTX. In both of the above pathways, SynB3‐PVGLIG‐PTX is targeted by MMP‐2, whereas only a limited amount of PTX is released due to the lack of MMP‐2 in normal brain tissue.

## 4 Conclusion

**Figure 10:**
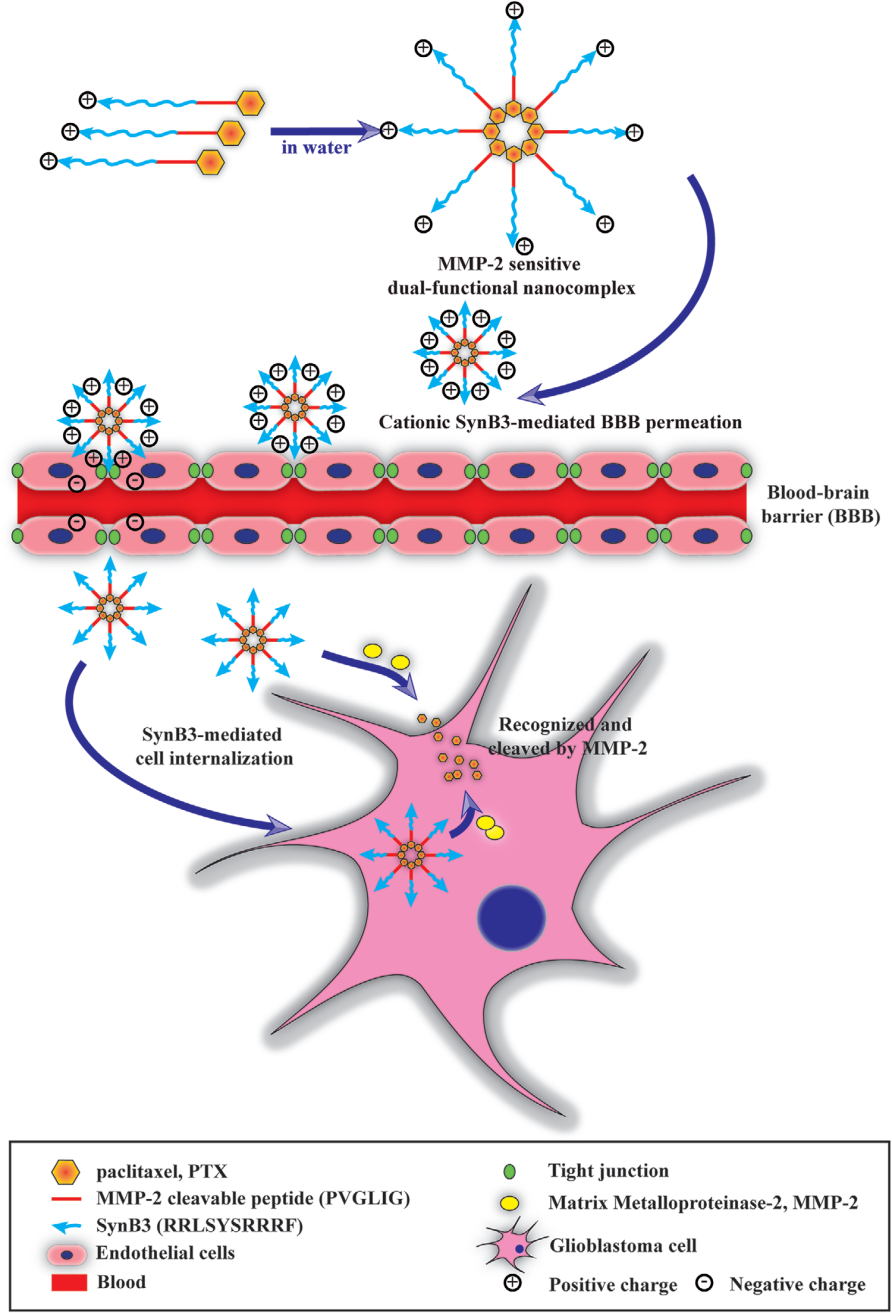
Schematic of drug delivery and release strategy of SynB3‐PVGLIG‐PTX. SynB3‐PVGLIG‐PTX is a novel dual‐functional nanocomplex drug delivery system following the strategy of PDCs.

In conclusion, we have designed a novel drug delivery system following the strategy of PDCs, in which two different functional peptides were combined to deal with drug delivery concerns of PTX in the treatment of GBM. The major conclusions from the study of the novel SynB3‐PVGLIG‐PTX nanocomplex were that (**Figure** **10**): 1) SynB3‐PVGLIG‐PTX exhibited a strong affinity with MMP‐2, and it could enhance water solubility by agglomerating to form a special structure with a positive charge; 2) a controlled release of PTX from SynB3‐PVGLIG‐PTX occurred upon cleavage of MMP‐2, implying that SynB3‐PVGLIG‐PTX has a specific cytotoxicity in GBM cells; 3) SynB3‐PVGLIG‐PTX can effectively inhibit the proliferation, migration, and invasion of GBM cells in vitro and in vivo. Furthermore, the inhibition rate of SynB3‐PVGLIG‐PTX was significantly higher than those of TMZ and PTX; and 4) the combined use of an MMP‐2‐sensitive peptide can be performed, and CPPs (SynB3) in a dual‐functional peptide enhanced BBB permeation and the glioma targeting effect of SynB3‐PVGLIG‐PTX, leading to high antitumor activity with low adverse effects during treatment.

## 5 Experimental Section

##### Protein Preparation for Molecular Docking

The crystal structure of MMP‐2 was obtained from the RCSB Protein Data Bank (PDB; https://www.rcsb.org/) with the code 1CK7.^[^ ^48^ ^]^ All ligands and water molecules were removed from the original PDBs. In order to perform molecular docking, the protein was prepared by supplementing the missing hydrogens and minimized with the force of the CHARMm force field using default parameters. All the preparation processes were implemented using the Discovery Studio software (DS v3.5; Accelrys, Inc., San Diego, CA, USA). Furthermore, the critical residues situated in the active sites of MMP‐2 were identified as ASP46, LEU89, GLU95, ARG98, Lys429, GLY216, GLU217, GLY218, GLN219, VAL220, TYR232, SER369, GLY371, LYS372, GLN393, GLU484, and LEU548. The amino acid residues interact with the ligand to perform pertinent biological functions.

##### Design and Preparation of PDC Candidates for Molecular Docking

To make the PDCs bioreversible conjugates that can be recognized by MMP‐2, the drugs always covalently bound to peptides with their ester, amide, disulfide, or hydrazine bond.^[^ ^49^ ^]^ In the present study, the high‐activity hydroxy group PTX (2′‐OH) could be coupled with the C‐terminal of the MMP‐2‐sensitive linker (PVGLIG) directly by an ester bond or linked with the N‐terminal of PVGLIG through a malonyl spacer adduct. In the next step, the CPPs with the BBB penetrating characteristic was linked to the other end of PVGLIG through an amide bond forming the final dual‐functional peptide‐PTX conjugate.^[^ ^20^, ^21^, ^22^ ^]^ Using these methods, ten conjugates were designed (Table S1, Supporting Information). All chemical structures of the ten conjugates were drawn on 2D sketcher. Thereafter, water molecules were discarded, missing hydrogens were added, and optimization was performed using CHARMm force on the Accelrys DS v3.5 (Accelrys, Inc., San Diego, CA, USA) for molecular docking.

##### Molecular Docking

In this study, molecular docking was performed for selecting the conjugate with the best affinity to MMP‐2 and predicting the docking mode between MMP‐2 and the conjugate. For assessing the affinity between MMP‐2 and different conjugates, molecular docking was implemented to appraise the precise docking score for each conjugate with MMP‐2. To do this, the Cdocker method with a set of default parameters available on the DS (Accelrys, Inc., San Diego, CA, USA) was used. The different conjugates were located and docked at the active site of the MMP‐2 crystal structure. The binding free energy between the conjugate and MMP‐2 was represented in the form of kcal mol^−1^ as the docking score.^[^ ^50^ ^]^ Based on the results, the complex with the lowest binding free energy for MMP‐2 was determined and selected for further examination.

##### Synthesis of SynB3‐PVGLIG‐PTX and Peptide

SynB3‐PVGLIG‐PTX was synthesized by Chinese Peptide Company (Hangzhou, China). The raw final product was purified by HPLC with a C_18_ reversed‐phase column. The mobile phase used in HPLC was a binary solvent system of water with 0.1% trifluoroacetic acid (solvent A) and acetonitrile/water (at a ratio of 80:20, v/v) with 0.09% trifluoroacetic acid (solvent B). The product was eluted at a flow rate of 1.0 mL min^−1^ by using a linear gradient from 47% B to 77% B in 20 min. HPLC analysis was performed by a 5 µL of injection volume. The ultraviolet wavelength for detection was set at 220 nm. The injection volume was 5 µL. The SynB3‐PVGLIG‐PTX product was obtained as a white powder at a yield of 87.9% (50 mg) and identified by mass spectrometry (MS; MW: 2855.2; Figure S4, Supporting Information), and by infrared spectroscopy (IR) and ^1^H NMR spectra (Figure S5, Supporting Information). In this study, all other conjugates with purity of 95% were synthesized by Chinese Peptide Company (Hangzhou, China), which were purified by HPLC and identified by MS.

##### Preparation of PDC Nanocomplex and NTA

SynB3‐PVGLIG‐PTX was dissolved in Dulbecco's phosphate buffered saline (PBS, pH 7.0; Gibco, Grand Island, NY) to prepare a solution at a 3 mg mL^−1^ concentration. The samples were filtered with a microporous membrane with a 0.22 µm PES low binding syringe‐driven filter unit (Millex GP, Millipore, Ireland) and equilibrated at room temperature. Then 50 µL of the solution was used every minute to carry out the NTA. The NTA measurements were performed with a Malvern Nanosight NS300 (Malvern Instruments, Worcestershire, UK) at room temperature. The dispersion samples were injected into the sample chamber using sterile syringes (BD Discardit II, New Jersey, USA) until the liquid reached the tip of the nozzle and was sonicated for 1 min automatically. During these experiments, video images were captured in the default mode. The mean size and SD of the particle was obtained and analyzed using the NTA 3.2 Dev Build 3.2.16 software.

##### Characterization of the SynB3‐PVGLIG‐PTX Nanocomplex

≈3 mg SynB3‐PVGLIG‐PTX was dissolved in PBS (1 mL) to prepare a nanoconjugate solution for this experiment. The mean PDI of SynB3‐PVGLIG‐PTX was determined using a DLS instrument (BI‐90Plus, Brookhaven Instruments Ltd., USA). A ZP analyzer (Zeta PALS, Brookhaven Instruments Ltd, USA) was applied to detect the ZP of SynB3‐PVGLIG‐PTX. All tests were repeated (*n* = 3) at 25 °C using a default setting to obtain the particle size, PDI, and ZP of the nanocomplex. The morphologies of the nanocomplex were then observed by TEM (HITACHI HT‐7700, Japan).

##### Release Study for SynB3‐PVGLIG‐PTX

A proteolysis experiment was conducted to ascertain whether PTX could be released from SynB3‐PVGLIG‐PTX upon cleavage of the MMP‐2 enzyme. The recombinant human MMP‐2 protein with 62.0 kDa molecular weight was purchased from PeproTech (Rocky Hill, NJ, USA). To observe the ability of cleavage in vitro, MMP‐2 was incubated with 4‐aminophenylmercuric acetate (7 × 10^−3^ m) at 37 °C for 1 h. After the activation process, SynB3‐PVGLIG‐PTX (2.5 mg mL^−1^) or SynB3‐PTX (2.5 mg mL^−1^) was added into MMP‐2 (5 ng µL^−1^). The reacting solution (50 µL) was then collected and analyzed through HPLC/MS. The spectra data were collected at 220 nm using gradient solvent conditions. BEH C18, 1.7 × 10^−6^ м, 100 × 2.1 mm column was used. The chromatographic conditions used for the analyses were: solvent A – 0.1% formic acid in water; solvent B – 0.1% formic acid in acetonitrile: 0–15 min: 2% MeCN, 15–18 min: 100% MeCN, 18–20 min: 2% MeCN, with a flow rate of 0.3 mL min^−1^.

##### Cell Lines and Cell Culture

The human GBM cell lines (U87MG and U251) were respectively obtained from the American Type Culture Collection (ATCC, Manassas, VA) and the China Academic Sinica Cell Repository (Shanghai, China) and cultured in accordance with the protocol of the supplier. In brief, all the cell lines were maintained in Dulbecco's Modified Eagle Medium (DMEM; Gibco, Grand Island, NY) supplemented with 10% fetal bovine serum (FBS; Biological Industries, Kibbutz Beit Haemek, Israel). Cells were incubated in a humidified incubator at 37 °C under 5% CO_2_ atmosphere.

##### Design and Transfection of siRNA

Homo‐MMP‐2 siRNA and nonsilencing siRNA were designed and synthesized by GenePharma (Suzhou, China). The siRNA sequences are shown in **Table** **4**. The transfection of siRNA was performed using the X‐tremeGENE siRNA transfection reagent (Roche, Mannheim, Germany) in accordance with the manufacturer's instructions. Before transfection, all the cell lines were inoculated at a density of 5 × 10^5^ cells per well in a 6‐well plate. After incubation overnight, the cells were washed with fresh DMEM medium without FBS. At the same time, the reagent of transfection was mixed with homo‐MMP‐2 siRNA or nonsilencing siRNA (50 × 10^−9^ м) for 15 min at a room temperature. The solution of the transfection complex was then added into the cell for 24 h. As a negative control group, a nonsilencing siRNA was transfected in the cell line in order to eliminate the influence of the transfection reagent.

**Table 4:** siRNA sequences used

Name		Sequence
Homo‐MMP‐2 siRNA	Sense	5′‐GCACCCAUUUACACCUACATT‐3′
	Antisense	5′‐UGUAGGUGUAAAUGGGUGCTT‐3′
Nonsilencing siRNA	Sense	5′‐UUCUCCGAACGUGUCACGUTT‐3′
	Antisense	5′‐ACGUGACACGUUCGGAGAATT‐3′

##### Western Blot Analysis

All cells were lysed using a RIPA lysis buffer (Solarbio, Beijing, China), supplemented by a complex of a protease inhibitor and a phosphatase inhibitor (1:100; Solarbio, Beijing, China) and phenylmethanesulfonylfluoride (PMSF; 1:100; Solarbio, Beijing, China). The concentrations of protein extracts were measured with the BCA protein assay kit (Solarbio, Beijing, China). The protein extracts were divided by a 10% sodium dodecyl sulfate‐polyacrylamide gel electrophoresis (SDS‐PAGE) and transferred to a PVDF membrane (Merck Millipore, Billerica, MA, USA). The membrane was sealed with 5% nonfat dry milk powder (Solarbio, Beijing, China) at room temperature for 2 h, and subsequently incubated overnight at 4 °C with the following primary antibodies: rabbit antihuman MMP‐2 (1:1000; Boster, Wuhan, China) and mouse antihuman GAPDH antibody (1:2000; Boster, Wuhan, China). Mouse antihuman GAPDH antibody was used as the loading control in this experiment. The membrane was subsequently washed with a PBS buffer containing 0.1% Tween‐20 for 10 min and followed by incubation with goat antirabbit or goat antimouse horse radish peroxidase (HRP)‐conjugated (1:2500; Santa Cruz, Dallas, Texas, USA) secondary antibody for 1 h at 25 °C. Blots of the immunocomplexes were colored using an enhanced chemiluminescence (ECL) reagent on a G: BOXiChemi XT chemiluminescence and fluorescence imaging system (Syngene, Cambrige, UK).

##### MTS Assay

To evaluate the antitumor activity of SynB3‐PVGLIG‐PTX, MTS (3‐(4, 5‐dimethylthiazol‐2‐yl)‐5‐(3‐carboxymethoxyphenyl)‐2‐(4‐sulfophenyl)‐2H‐tetrazolium) assay was performed on the U87MG and U251 cell lines using the following protocols. All the cell lines and MMP‐2‐siRNA‐treated transfected cells lines were seeded at a density of 3 × 10^3^ cells per well in 96‐well plates for 24 h, and subsequently treated with free TMZ (Sigma‐Aldrich, St Louis, MO, USA), PTX (≥98% by HPLC; Sigma‐Aldrich, St Louis, MO, USA), SynB3‐PTX and SynB3‐PVGLIG‐PTX at a concentration of 25 × 10^−6^, 50 × 10^−6^, 75 × 10^−6^, and 100 × 10^−6^ м for 4, 24, and 48 h, respectively. The cells treated with PBS and 100 × 10^−6^ м SynB3‐PVGLIG were used as the control and the dual‐functional peptide control, respectively. After treatment, the cells were washed with PBS three times and subsequently incubated with 20 µL Cell Titer 96AQeuous One Solution Reagent (Promega, Madison, WI, USA) for 2 h at 37 °C. The absorbance was measured on a Synergy microplate reader (BioTek Instruments, Winooski, VT) at 490 nm. All the data were obtained from three independent experiments performed in three wells. The absorbance of cells in the control group was taken as 100% viability, and the inhibition rate of cell viability was counted by using the following equation: % Inhibition rate = (Absorbance_control_ − Absorbance_test_)/(Absorbance_control_ − Absorbance_blank_) *×* 100. The IC50 value showed the concentration of different drugs that were required to result in 50% inhibition of cell proliferation activity, and was calculated by linear regression.

##### Transwell Assay

Both migration and invasion activities of cells were tested with the Transwell chamber (Merck Millipore, Billerica, MA, USA). For detecting the invasion of the cells, the bottom surface of the membrane in each Transwell chamber was precoated with 50 µL‐diluted Matrigel (BD Bioscience, Mountain View, CA, USA). A total of 3 × 10^4^ cells were suspended in 200 µL DMEM with 50 × 10^−6^ м TMZ, 50 × 10^−6^ м SynB3‐PVGLIG, 50 × 10^−6^ м PTX, and 50 × 10^−6^ м SynB3‐PVGLIG‐PTX, respectively. The cell suspensions were then transferred to the upper Transwell chamber, and 500 µL DMEM with 10% FBS was added in the bottom well. After 24 h incubation in a 37 °C humidified incubator with 5% CO_2_, the cells adhering to the filter surface of each chamber were fixed with 4% paraformaldehyde (PFA; Sigma‐Aldrich St. Louis, MO, USA) and stained with 0.1% crystal violet (Solarbio, Beijing, China). The cells at the bottom side of the chamber were counted in three randomly selected 200 × microscopic fields under a DM6000B microscope (Leica, Wetzlar, Germany).

##### Orthotopic U87MG Tumor Transplantation in Nude Mice and Bioluminescent Imaging In Vivo

A total of 20 six‐week‐old BALB/C athymic nude mice were bought from the National Laboratory Animal Center in Beijing. All the animal experiment procedures were approved by the Institutional Animal Care and Use Committee of TMUGH. For the xenograft nude mice assay, all the nude mice were anesthetized with an intraperitoneal injection of 10% chloral hydrate. On day 0, U87MG cells (5 × 10^5^) in 3 µL PBS were injected into the right cerebral hemisphere of anesthetized nude mice. The specific coordinate of the injection was between bregma and posterior fontanelle, 1 mm lateral to the bregma, and 3‐mm deep from the dura mater. IVIS Lumina Imaging System (PerkinElmer, Waltham, MA) was used to monitor and analyze the presence, location, and growth volume of tumors in xenograft nude mice. All images were acquired under an identical illumination setting and displayed in the form of p s^−1^ cm^−2^ sr^−1^ that represents the volume of the tumor.

##### Antitumor Activity In Vivo

On day 7, the radiance of the tumor in vivo approximately reached 0.9 × 10^5^–1.5 × 10^5^ p s^−1^ cm^−2^ sr^−1^ measured using the bioluminescent imaging method described above. The mice were randomly divided into four groups (five mice in each group), with each group respectively treated with physiological saline, TMZ, PTX, and SynB3‐PVGLIG‐PTX. TMZ was administered at a dose of 40 mg kg^−1^ (five times a week) by gavage, while the physiological saline, PTX (15 mg kg^−1^, twice a week), and SynB3‐PVGLIG‐PTX (15 mg kg^−1^, twice a week) were given via tail vein injection. All mice were treated for five consecutive weeks. The growth of the xenograft tumor was monitored using the bioluminescent imaging on the 7th, 14th, 21st, and 28th day after implantation of U87MG cells. The BLI of the cerebral tumor in the control group was taken as 100% tumor growth capacity, and the tumor inhibition rate was counted by using the following equation: % tumor inhibition rate = (1 − BLI_test_ /BLI_control_) *×* 100. The health of the mice was monitored daily, and the weights of mice were measured every other day until death. At day 35, all the mice were sacrificed and the brains with tumors were excised and weighed.

##### H&E Staining

Brain tissue samples were fixed in 3.7% formaldehyde solution, dehydrated, and embedded in paraffin, and cut into sections around 5 µm thick according to standard procedures. For further histological analysis, the paraffin sections were subjected with H&E staining. Images of brain sections stained with H&E were obtained under a DM6000B fluorescent microscope (Leica, Wetzlar, Germany). For every section, three fields were randomly selected and observed.

##### IHC Staining

In order to evaluate the antitumor efficacy of different drugs, IHC staining was performed with a rabbit antihuman Ki‐67 antibody as the primary antibody (Millipore, Billerica, MA). Briefly, endogenous peroxidase activity was blocked in the deparaffinized sections by incubating in 0.3% (v/v) hydrogen peroxide for 15 min at room temperature, after which the sections were incubated in normal goat serum for further blockage. Thereafter, the sections were washed with a tris‐buffered saline (TBS) solution and then incubated with the primary antibody at 4 °C overnight. Subsequently, the sections were incubated gradually with secondary antibody labeled with HRP. The sections were then colored with 3, 3′‐diaminobenzidine tetrahydrochloride (DAB; Sigma‐Aldrich, St Louis, MO, USA). Hematoxylin was used for counterstaining. Finally, the sections were dehydrated and mounted with neutral resin, and images were observed and captured with a microscope. The positive cell numbers and total cell numbers were counted in six randomly selected 400 × microscopic fields using the Image Pro Plus 5.0 software (Leica, Wetzlar, Germany). The labeling index (%; LI) was calculated as the percentage ratio of positive cell numbers to total cell numbers.

##### Real‐Time Fluorescence Imaging and Biodistribution Analysis

Two groups of mice (*n* = 18 per group) were treated with Cy5‐labeled SynB3‐PVGLIG‐PTX and Cy5‐labeled PTX (5.25 µmol/5 mL kg^−1^), respectively, in which the dose was equivalent to the effective dose of SynB3‐PVGLIG‐PTX (15 mg/5 mL kg^−1^) in the antitumor in vivo experiment. An untreated group was administered with physiological saline as a control. Real‐time fluorescence images were captured and analyzed using the IVIS Lumina Imaging System (PerkinElmer, Waltham, MA). The results were used to evaluate the real‐time tissue distribution of SynB3‐PVGLIG‐PTX in vivo. At 0.5, 2, 4, 12, 24 and 48 h after administration, 3 mice were taken from each group to capture real‐time fluorescence images of the tumor and drug distribution, and the images were analyzed by the IVIS Lumina Imaging System. The excitation and emission quantitative wavelengths used were 640 nm and 700 nm, respectively. At the end of each experiment, animals were euthanized and their brains, lungs, hearts, livers, and kidneys were harvested and further tested by the IVIS Lumina imaging system to determine the drug distribution in tissue from each organ.

##### Cerebral Distribution Studies

PTX levels in the mouse brains were measured by the HPLC/MS method. Twenty nude mice with orthotopic U87MG transplantation were assigned at random into two groups: a SynB3‐PVGLIG‐PTX group (5.25 µmol/5 mL kg^−1^, i.v.; 15 mice) and a control group (PBS, i.v.; 5 mice). After injection of SynB3‐PVGLIG‐PTX, 5 mice were selected at 2, 4, and 12 hours, respectively, and sacrificed. Tumor‐free brain tissue as well as the tumor were isolated and weighed separately. The tissue was placed on a 35 mm dish and washed with PBS on ice. The tissue was then cut into small pieces with a surgical scissor and transferred into a 1.5 mL centrifuge tube. Appropriate amounts of 50% methanol were added into the tube to prepare the tissue suspension (0.2 g mL^−1^). The tissue suspensions were thoroughly homogenized by an ultrasonic cell disrupter. Thereafter, the tissue homogenates were centrifuged at 10 000 × g for 30 min. The precipitate was removed, and the supernatant was collected for further content analysis by HPLC/MS. In HPLC/MS analysis, whole brains of mice in the control group were taken as a blank sample, and 100 µg mL^−1^ PTX was added into another blank sample to prepare the positive control. The drug response values were obtained and analyzed.

##### Statistical Analysis

All data in this study were analyzed using Graphpad Prism version 6.00 for Windows (GraphPad Software, La Jolla California USA) and are presented as mean ± standard deviation (s.d.). of three independent experiments. Statistical significance was analyzed by using analysis of 1‐way and 2‐way variance (ANOVA), followed by Tukey post‐hoc or Dunn's post‐hoc tests, and assigned at *p < 0.05, **p < 0.01, or ***p < 0.001. For the study of Kaplan‐Meier analysis of overall survival in vivo, differences were tested by log‐rank test, and considered significant at *p < 0.05, and **p < 0.01.

## Conflict of Interest

The authors declare no conflict of interest.

## Supporting information

Supporting Information
