# Correction to “Improved Antiglioblastoma Activity and BBB Permeability by Conjugation of Paclitaxel to a Cell‐Penetrative MMP‐2‐Cleavable Peptide”

**DOI:** 10.1002/advs.202510150

**Published:** 2025-10-05

**Authors:** Dan Hua, Lida Tang, Weiting Wang, Shengan Tang, Lin Yu, Xuexia Zhou, Qian Wang, Cuiyun Sun, Cuijuan Shi, Wenjun Luo, Zhendong Jiang, Huining Li, Shizhu Yu

Adv. Sci. 2021, Feb. 8(3):2001960

https://doi.org/10.1002/advs.202001960

In the Supporting Information of the original publication, the independent image representing the migration in the U251 without the MMP‐2 siRNA group was incorrectly presented in Figure S2 (Supporting Information). The correct image should be as follows:

 
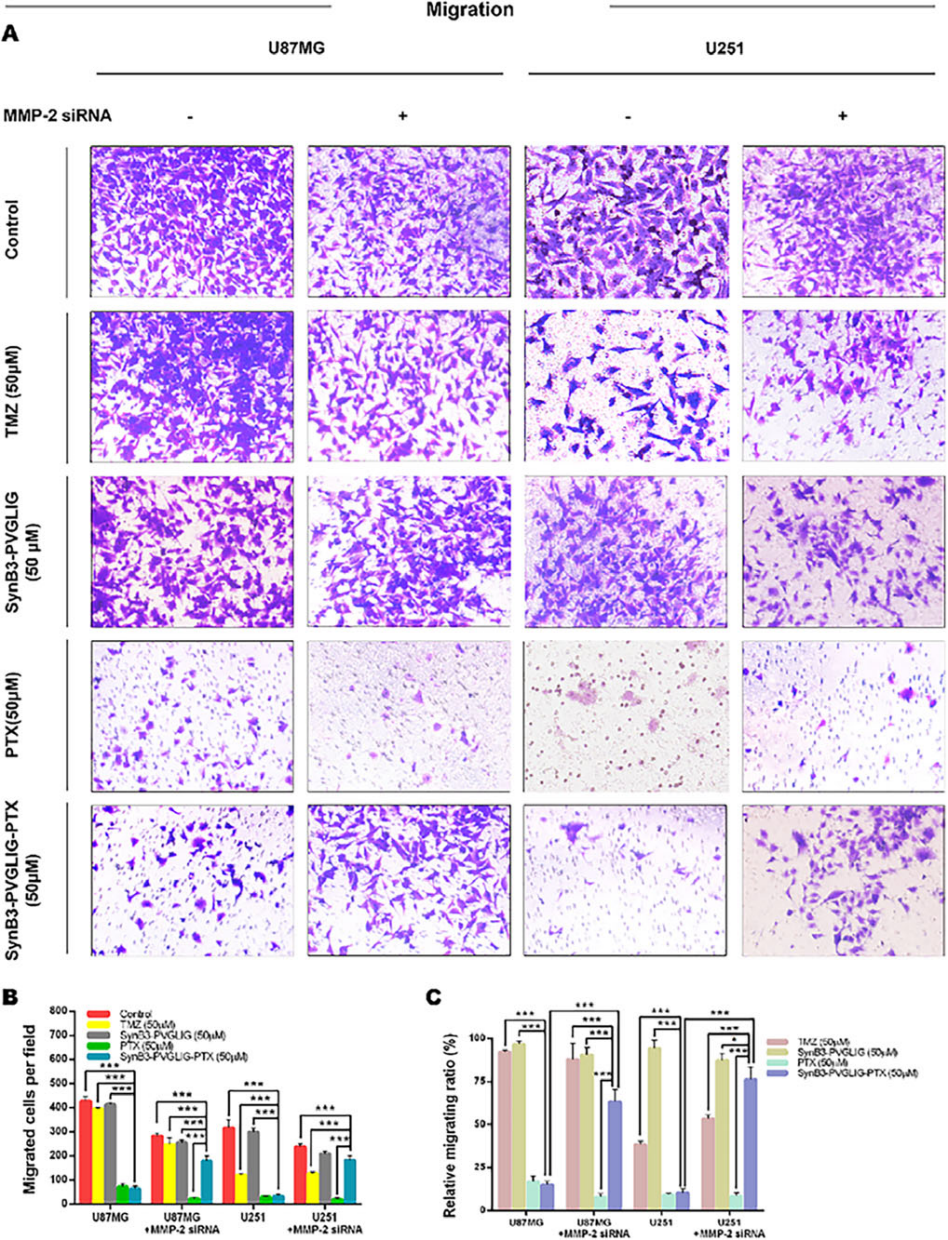


We apologize for this error.

