# Supplementary material for: Improved Antiglioblastoma Activity and BBB Permeability by Conjugation of Paclitaxel to a Cell‐Penetrative MMP‐2‐Cleavable Peptide
Source: Adv Sci (Weinh). 2020 Dec 21;8(3):2001960. doi: 10.1002/advs.202001960 (PMC7856885; doi:10.1002/advs.202001960)
Supplement: Supplementary file 1 — Supporting Information [file ADVS-8-2001960-s001.pdf]

WILEY-VCH

© 2020 Wiley-VCH GmbH

ADVANCED SCIENCE
Open Access

Supporting Information

for *Adv. Sci.,* DOI: 10.1002/advs.202001960

Improved Anti-glioblastoma Activity and BBB Permeability by Conjugation of Paclitaxel to a Cell-penetrative MMP-2-cleavable Peptide.

*Dan Hua, Lida Tang, Weiting Wang, Shengan Tang, Lin Yu, Xuexia Zhou, Qian Wang, Cuiyun Sun, Cuijuan Shi, Wenjun Luo, Zhendong Jiang, Huining Li, and Shizhu Yu**

Supporting Information

Improved Anti-glioblastoma Activity and BBB Permeability by Conjugation of Paclitaxel to a Cell-penetrative MMP-2-cleavable Peptide.

*Dan Hua, Lida Tang, Weiting Wang, Shengan Tang, Lin Yu, Xuexia Zhou, Qian Wang, Cuiyun Sun, Cuijuan Shi, Wenjun Luo, Zhendong Jiang, Huining Li, and Shizhu Yu**

Table S1. Ten PDCs designed in this study.

ID	Linker	CPPs	PDCs
C1		pVEC	**PTX-2'OH-malonyl**-PVGLIG-**LLIILRRRIRKQAHAHSK**[a), b), c), d)]
C2		TP10	**PTX-2'OH-malonyl**-PVGLIG-**AGYLLGKINLKALAALAKKIL**
C3	Malonyl	TP10-2	**PTX-2'OH-malonyl**-PVGLIG-**AGYLLGKINLKPLAALAKKIL**
C4		SynB3	**PTX-2'OH-malonyl**-PVGLIG-**RRLSYSRRRF**
C5		Tat 47-57	**PTX-2'OH-malonyl**-PVGLIG-**YGRKKRRQRRR**
N1		pVEC	**LLIILRRRIRKQAHAHSK**-PVGLIG-**2'OH-PTX**
N2		TP10	**AGYLLGKINLKALAALAKKIL**-PVGLIG-**2'OH-PTX**
N3	No	TP10-2	**AGYLLGKINLKPLAALAKKIL**-PVGLIG-**2'OH-PTX**
N4		SynB3	**RRLSYSRRRF**-PVGLIG-**2'OH-PTX**
N5		Tat 47-57	**YGRKKRRQRRR**-PVGLIG-**2'OH-PTX**

[a)] The nomenclature of PTX-2'OH refers to that 2'-OH group of PTX was used to react with the peptide for forming the PDCs.

[b)] Underlined residues are the sequence of the MMP-2 sensitive peptide.

[c)] The group indicated in red was the linker between PTX and peptide.

[d)] The green amino acids were the CPPs which formed the nanocarrier.

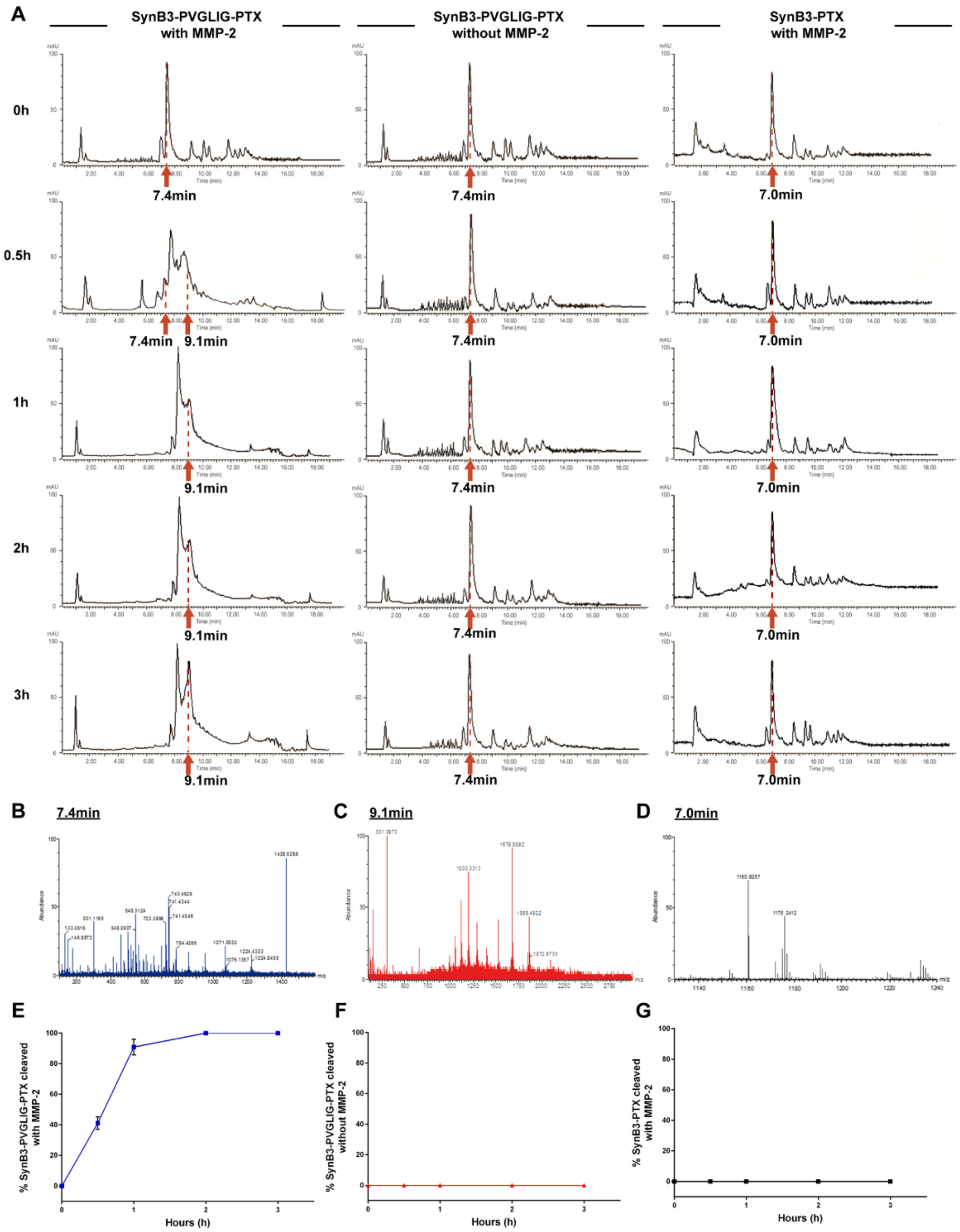

Figure S1. The hydrolysis of SynB3-PVGLIG-PTX and SynB3-PTX with or without MMP-2 *in vitro*. (A) Left: HPLC chromatograms of MMP-2 digested SynB3-PVGLIG-PTX after 0.5, 1, 2 and 3 h, observed at 220 nm; retention times t_R = 7.4 and 9.1 min for

SynB3-PVGLIG-PTX and PVG-PTX (thick dotted line) respectively. Middle: HPLC results of SynB3-PVGLIG-PTX without MMP-2 (220 nm); t_R = 7.4 min for SynB3-PVGLIG-PTX. Right: HPLC results (220 nm) after SynB3-PTX was incubated with MMP-2 for 0.5, 1, 2 and 3 h; the retention time tR of SynB3-PTX was 7.0 minutes. **(B-D)** The MS results. At 7.4 min, ESIMS m/z 1428.53 $[1/2M+H]^+$, SynB3-PVGLIG-PTX calcd. At 9.1 min, ESIMS m/z 1200.33 $[M+Na]^+$, PVG-PTX calcd, and ESIMS m/z 1679.89 $[M+H]^+$, SynB3-LIG calcd. At 7.0 min, ESIMS m/z 1160.83 $[1/2M+H]^+$, SynB3-PTX calcd. **(E-F)** The cleavability of SynB3-PVGLIG-PTX (2.5 mg/ml) by MMP-2 was measured by HPLC/MS after it was incubated with 5 ng/µl MMP-2 (blue line and point) or without MMP-2 (red line and triangle) for 0.5, 1, 2, 3 h at 37°C. **(G)**The cleavability of SynB3-PTX (2.5 mg/ml) by 5 ng/µl MMP-2 at 0.5, 1, 2, 3 h at 37°C was measured by HPLC/MS (black line and point).

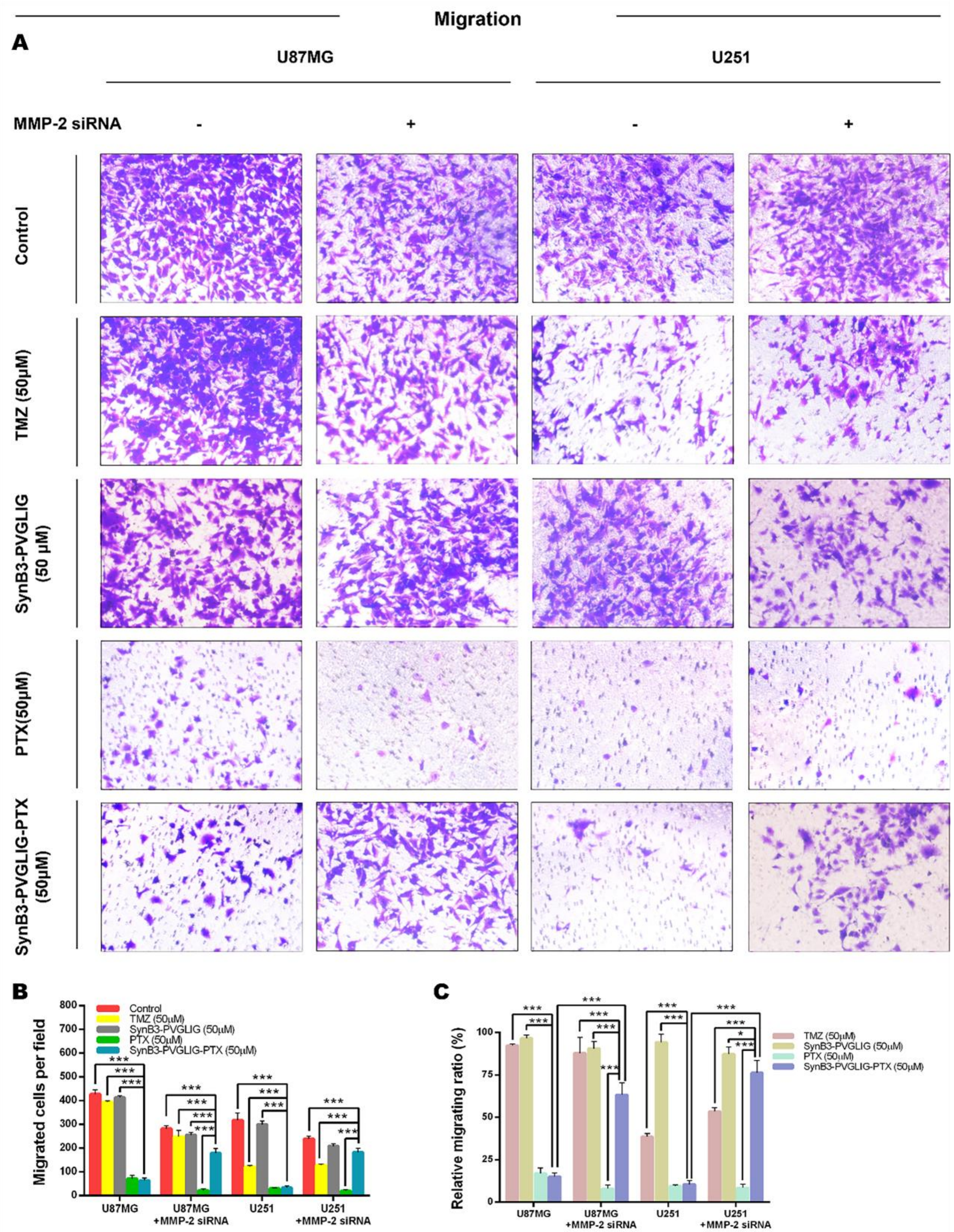

Figure S2. Effect of SynB3-PVGLIG-PTX on migration of different cell lines by Transwell assay. (A) The U87MG, MMP-2 siRNA-transfected U87MG, U251, and MMP-2 siRNA-transfected U251 were treated with TMZ, SynB3-PVGLIG, PTX and

SynB3-PVGLIG-PTX at the same dose (50 μM) for 24 h. Representative images of migrated cells in different groups are shown. The original magnification in this study was ×200. **(B)** The number of migrated cells per field. All data are presented as mean ± SD, n=3. Compared with SynB3-PVGLIG-PTX group, ***P<0.001 by 2-way ANOVA with Tukey's post-hoc. **(C)** The relative migration rate was presented as a percentage with the value of cells in control group being 100%. All data are expressed as mean ± SD, n=3. Compared with SynB3-PVGLIG-PTX group, *P<0.05, ***P<0.001 by 2-way ANOVA with Tukey's post-hoc.

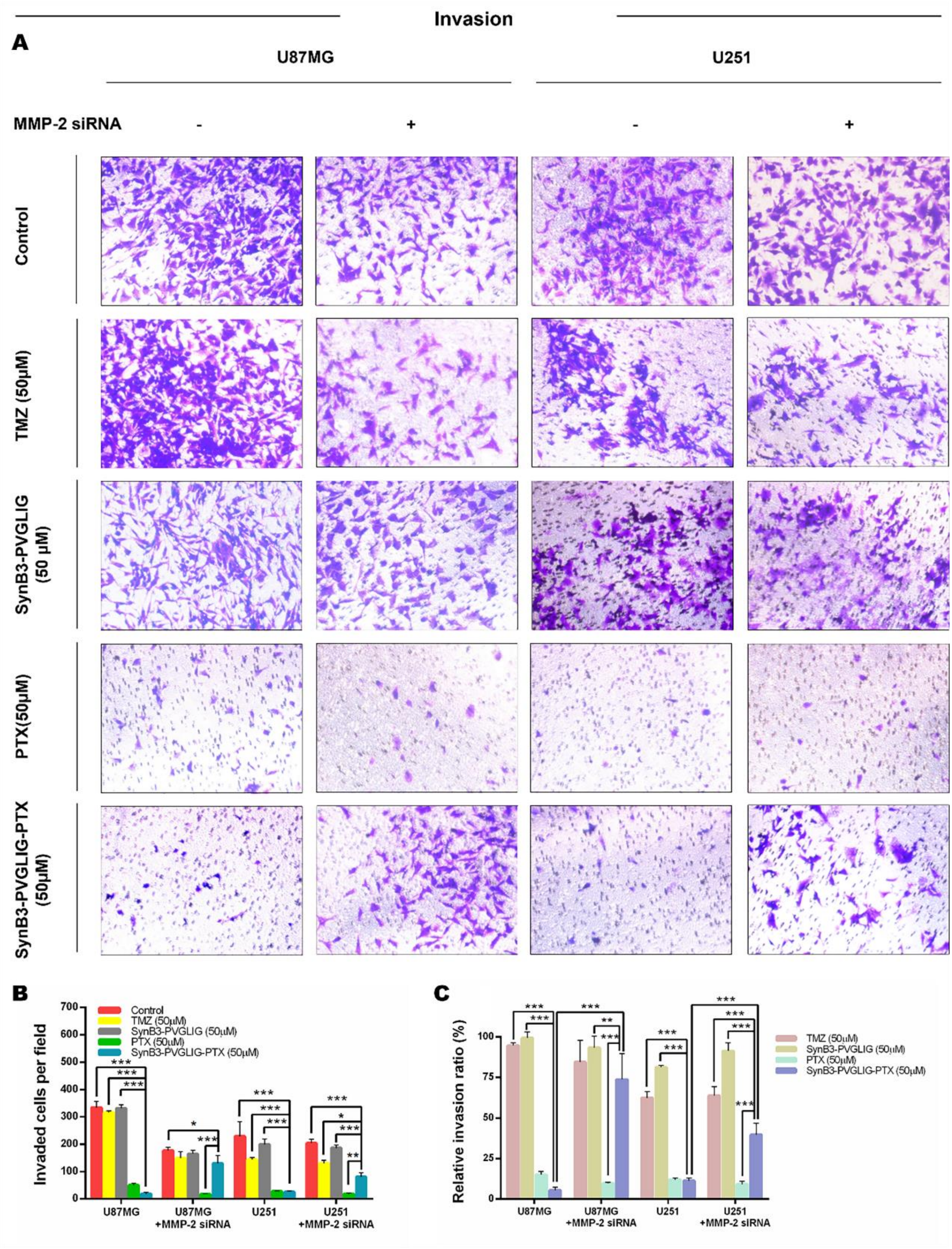

Figure S3. Effect of SynB3-PVGLIG-PTX on invasion of different cell lines by Transwell assay. (A) The U87MG, MMP-2 siRNA-transfected U87MG, U251, and MMP-2 siRNA-transfected U251 were treated with TMZ, SynB3-PVGLIG, PTX and

SynB3-PVGLIG-PTX at the same dose (50 μM) for 24 h. Representative images of invaded cells in different groups are shown. The original magnification in this study was ×200. **(B)** The number of invaded cells per field. All data are presented as mean ± SD, n=3. Compared with SynB3-PVGLIG-PTX group, **P*<0.05, ***P*<0.01, ****P*<0.001 by 2-way ANOVA with Tukey's post-hoc. **(C)** The relative invasion rate was presented as a percentage with the value of cells in control group being 100%. All data are expressed as mean ± SD, n=3. Compared with SynB3-PVGLIG-PTX group, ***P*<0.01, ****P*<0.001 by 2-way ANOVA with Tukey's post-hoc.

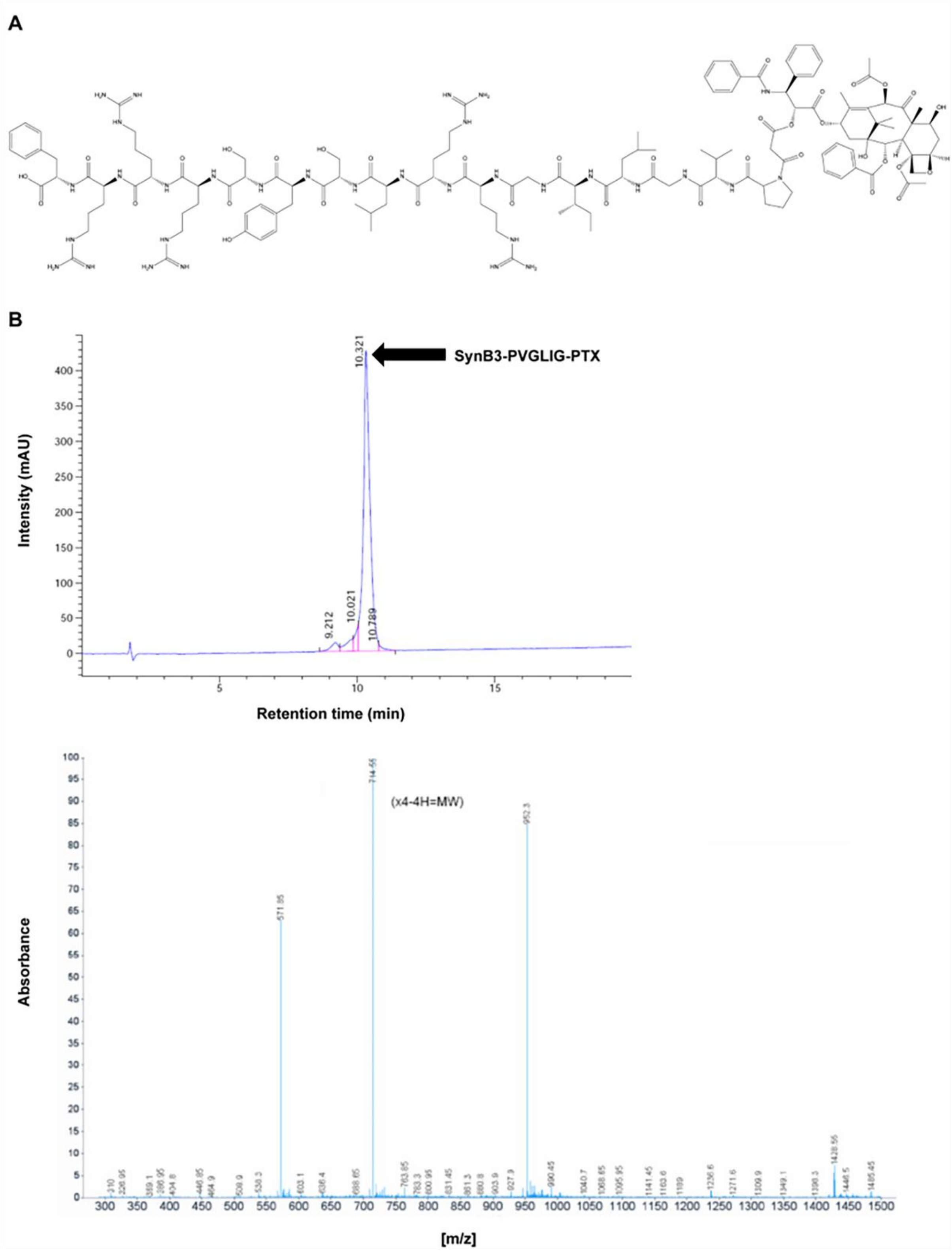

Figure S4. The identification of SynB3-PVGLIG-PTX. (A) The structure of SynB3-PVGLIG-PTX. **(B)** HPLC-MS analysis of synthetic product. SynB3-PVGLIG-PTX, white powder; yield: 87.9% (65 mg). ESI-MS: m/z 714.55 $[1/4M+H]^+$.

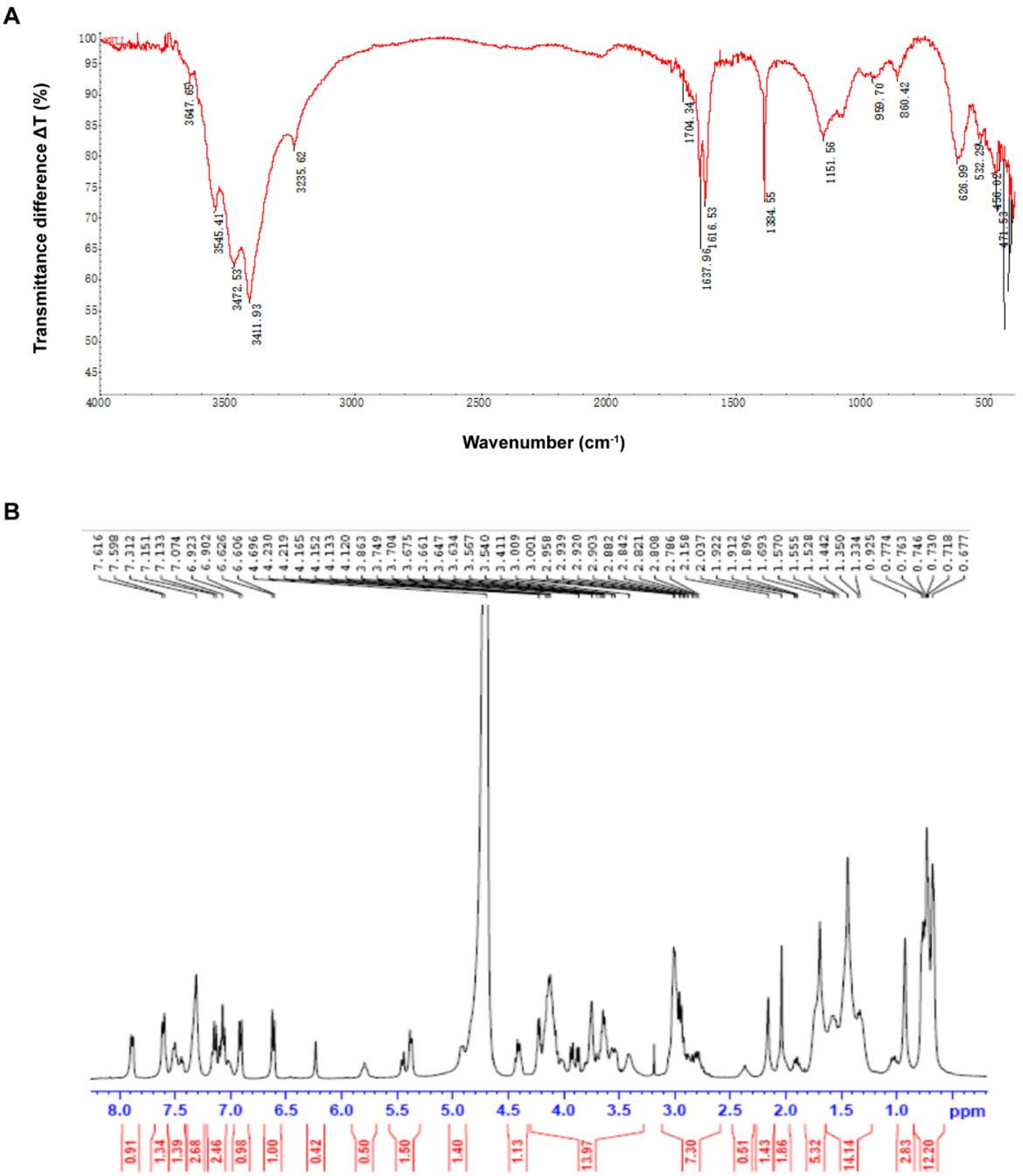

Figure S5. IR and ^{1}H NMR spectra of SynB3-PVGLIG-PTX. (A) IR (KBr) νmax (cm^{-1}): 3545, 3473, 3412, 3236, 1638, 1617, 1385, 1151. **(B)** ^{1}H NMR (400MHz, D_2O): δ 7.90 (d, *J* = 7.2Hz, 2H), 7.60 (d, *J* = 7.2Hz, 2H), 7.45 (m, 4H), 7.31 (m, 6H), 7.15 (m, 2H), 7.07 (m, 4H), 6.91 (d, *J* = 8.2Hz, 2H), 6.61 (d, *J* = 8.2Hz, 2H), 6.24 (br s, 1H), 5.79 (t, *J* = 8.0Hz, 1H), 5.45 (d, *J* = 8.5Hz, 1H), 5.38 (m, 2H), 4.92 (m, 1H), 3.41-4.44 (m, 36H), 2.70-3.01 (m, 17H), 2.37 (m, 1H), 2.16 (s, 3H), 2.04 (s, 3H), 1.89 (m, 2H), 1.69 (s, 3H), 1.33-1.60 (m, 23H), 0.93 (s, 6H), 0.67-0.77 (m, 30H).

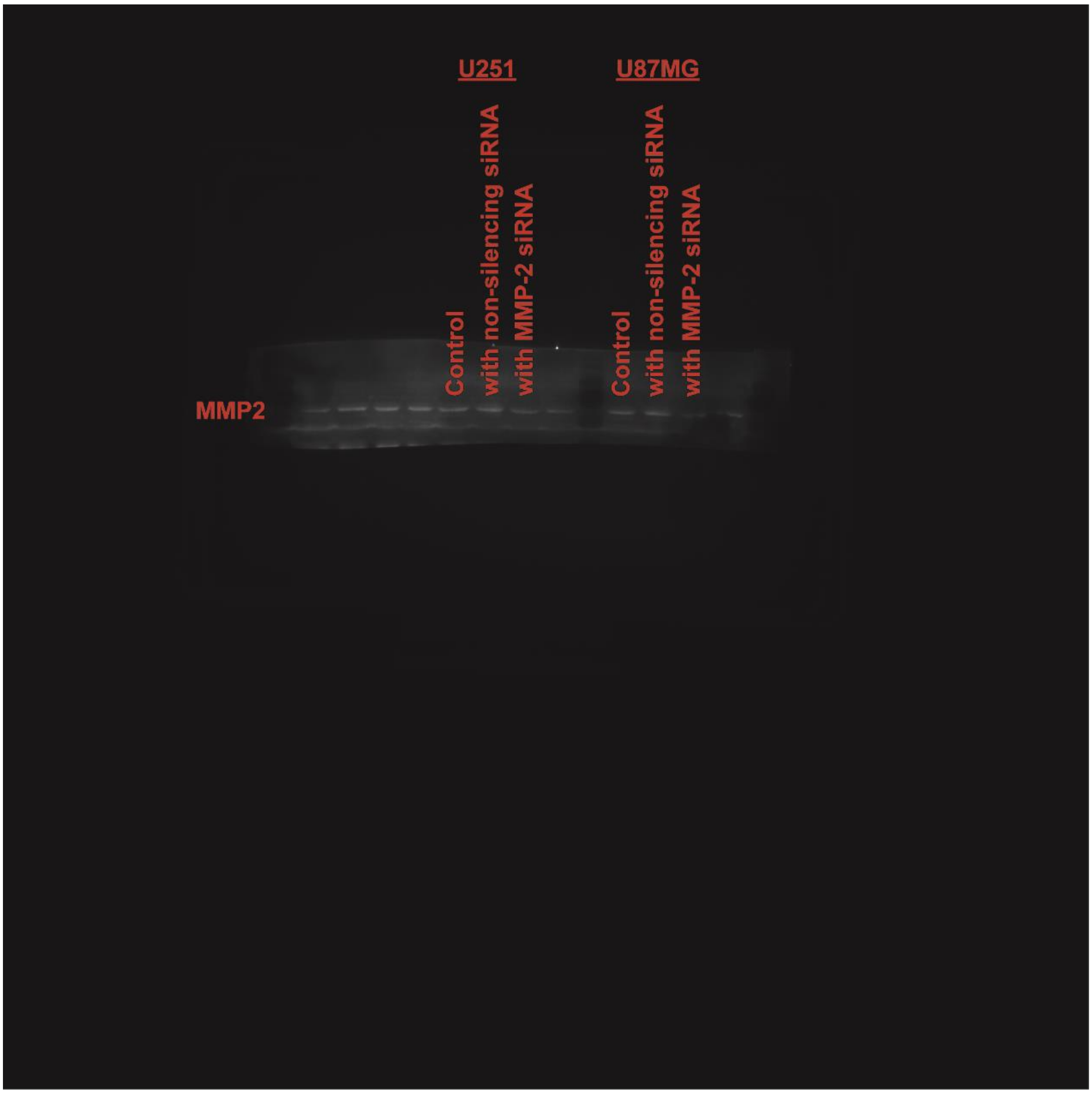

Figure S6. Original images of western blot with anti-MMP-2 in different protein extracts.

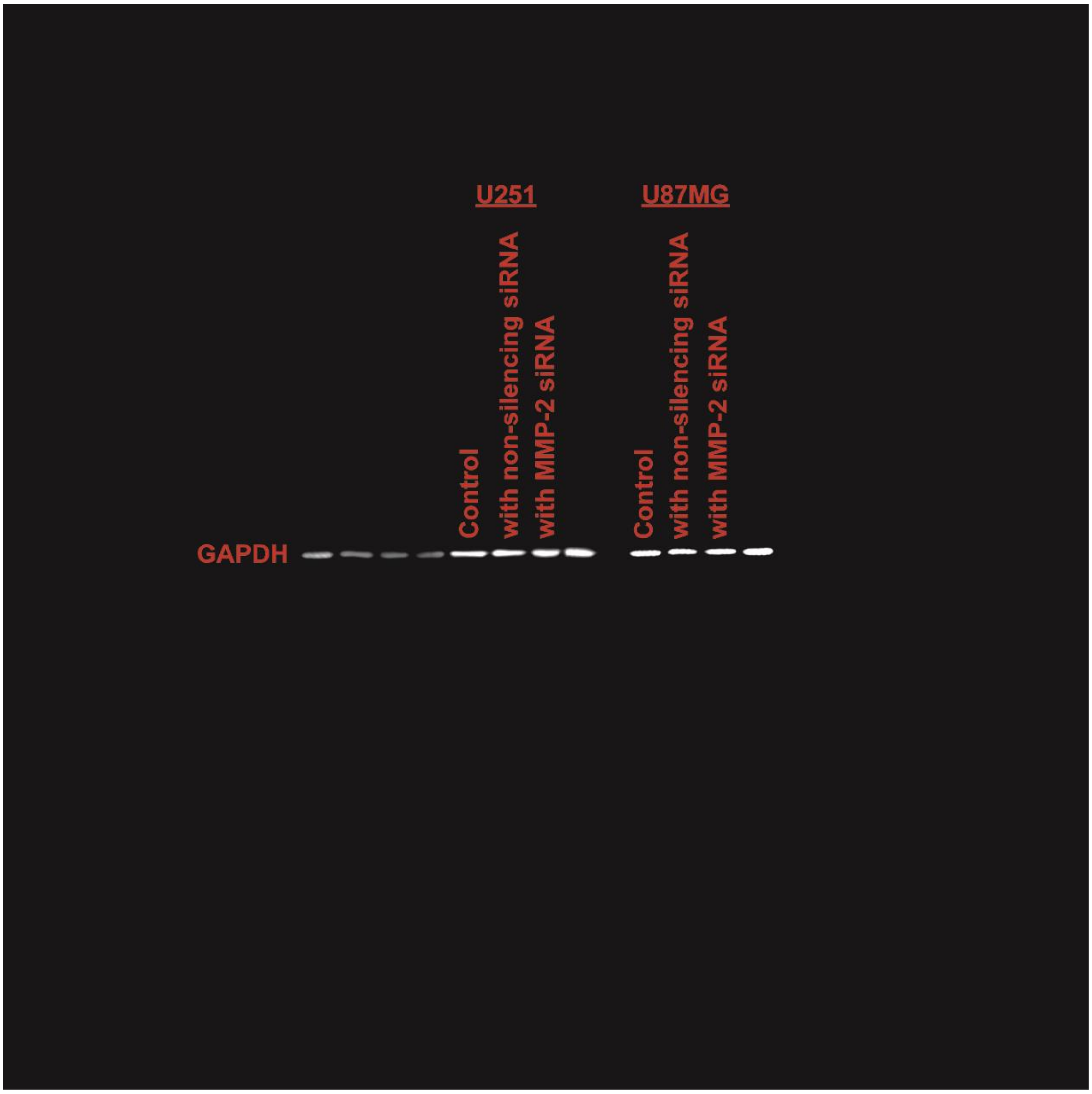

Figure S7. Original images of western blot with anti-GAPDH in different protein extracts.
